# The RNA-binding protein Celf1 post-transcriptionally regulates p27^Kip1^ and Dnase2b to control fiber cell nuclear degradation in lens development

**DOI:** 10.1371/journal.pgen.1007278

**Published:** 2018-03-22

**Authors:** Archana D. Siddam, Carole Gautier-Courteille, Linette Perez-Campos, Deepti Anand, Atul Kakrana, Christine A. Dang, Vincent Legagneux, Agnès Méreau, Justine Viet, Jeffrey M. Gross, Luc Paillard, Salil A. Lachke

**Affiliations:** 1 Department of Biological Sciences, University of Delaware, Newark, DE, United States of America; 2 Institut de Génétique et Développement de Rennes, Université de Rennes 1, CNRS UMR6290, Rennes, France; 3 Instituto Tecnológico de Costa Rica, Cartago, Costa Rica; 4 Department of Molecular Biosciences, University of Texas, Austin, TX, United States of America; 5 Center for Bioinformatics and Computational Biology, University of Delaware, Newark, DE, United States of America; 6 Department of Ophthalmology, Louis J. Fox Center for Vision Restoration, University of Pittsburgh School of Medicine, Pittsburgh, PA, United States of America; University of Kentucky, UNITED STATES

## Abstract

Opacification of the ocular lens, termed cataract, is a common cause of blindness. To become transparent, lens fiber cells undergo degradation of their organelles, including their nuclei, presenting a fundamental question: does signaling/transcription sufficiently explain differentiation of cells progressing toward compromised transcriptional potential? We report that a conserved RNA-binding protein Celf1 post-transcriptionally controls key genes to regulate lens fiber cell differentiation. *Celf1*-targeted knockout mice and *celf1*-knockdown zebrafish and *Xenopus* morphants have severe eye defects/cataract. Celf1 spatiotemporally down-regulates the cyclin-dependent kinase (Cdk) inhibitor p27^Kip1^ by interacting with its 5’ UTR and mediating translation inhibition. Celf1 deficiency causes ectopic up-regulation of p21^Cip1^. Further, Celf1 directly binds to the mRNA of the nuclease Dnase2b to maintain its high levels. Together these events are necessary for Cdk1-mediated lamin A/C phosphorylation to initiate nuclear envelope breakdown and DNA degradation in fiber cells. Moreover, Celf1 controls alternative splicing of the membrane-organization factor beta-spectrin and regulates F-actin-crosslinking factor Actn2 mRNA levels, thereby controlling fiber cell morphology. Thus, we illustrate new Celf1-regulated molecular mechanisms in lens development, suggesting that post-transcriptional regulatory RNA-binding proteins have evolved conserved functions to control vertebrate oculogenesis.

## Author summary

Besides associated with aging, the eye disease cataract can occur early in life because of defects in lens development. Lens fiber cells degrade their nuclei to achieve lens transparency, which poses a fundamental question: how is differentiation regulated in a cell progressing toward compromised transcriptional potential? We demonstrate that this is achieved by distinct post-transcriptional gene expression control mechanisms mediated by a conserved RNA-binding protein Celf1. Celf1 regulates key factors required for normal nuclear degradation and cell morphology of fiber cells by controlling abundance of target mRNAs and/or their translation into protein. These data identify new Celf1-regulated molecular mechanisms necessary for lens transparency and suggest that conserved post-transcriptional regulatory RNA-binding proteins have evolved to control eye development in vertebrates.

## Introduction

Interaction of RNA-binding proteins (RBPs) with target mRNA is necessary for every aspect of its control, including its processing, export, localization, stability, and translation into protein [1]. These events are collectively defined as post-transcriptional control of gene expression and are essential to determine the proteome of a cell. While the role of transcription factors in vertebrate organogenesis is established–for example, a detailed transcriptional regulatory network governing lens development is now derived [2]–that of RBPs, functioning in post-transcriptional gene expression control, is not well defined [3]. This represents a significant knowledge gap, especially considering that vertebrate genomes encode similar numbers of transcription factors and RBPs [4].

Evolution of the ocular lens has enabled high-resolution vision in animals, and its development involves precise spatio-temporal control of gene expression that drives the formation of a transparent tissue containing anteriorly localized epithelial cells and posteriorly localized terminally differentiated fiber cells, which contribute to the bulk of the tissue [2,5]. To achieve transparency, lens fiber cells elongate, produce high amounts of refractive proteins called crystallins, and remove their organelles. Because they lose their nuclei, and with it their transcription potential, a fundamental question in ocular lens development is whether signaling and transcription are sufficient to explain the regulation of fiber cell differentiation. One hypothesis is that post-transcriptional regulatory mechanisms may have evolved to control these differentiation events as they provide additional layers of gene expression control [6,7,3]. However, the significance of post-transcriptional regulation, especially mediated by RBPs, is not characterized in lens development. Here we used a bioinformatics tool *iSyTE* (integrated Systems Tool for Eye gene discovery) to identify a conserved RBP Celf1 (CUGBP, Elav-like family member 1; also known as Cugbp1) as a new post-transcriptional regulator of lens development, and by applying a variety of cellular, molecular and animal model approaches elucidate the detailed molecular mechanism of Celf1-based post-transcriptional control in the lens. Celf1 is shown to directly bind to specific mRNAs through its three RNA recognition motifs (RRMs) and control their distinct post-transcriptional fates namely, alternative splicing, stability and translation [8,9]. Celf1 is well conserved at the amino acid level differing in only two amino acids between mouse and human, and importantly the three RRMs are highly conserved across vertebrates, suggesting its functional conservation in vertebrates [8–10]. Celf1 deficiency in mouse causes spermatogenesis defects [11] and its mis-regulation is associated with myotonic dystrophy in human [12]. Further, in cardiomyocytes, Celf1 is known to control alternative splicing [13]. We now provide new advances into how Celf1-mediated distinct post-transcriptional mechanisms spatiotemporally control the proteome of differentiating fiber cells in lens development.

Here, we show that Celf1 is essential for eye development in diverse vertebrates such as fish (zebrafish), amphibians (*Xenopus*) and mammals (mouse). Characterization of lens-specific *Celf1* deletion mice by various phenotypic analyses reveals embryonic-onset defects involving abnormal cell morphology and defective degradation of nuclei that culminate into cataract. Further, molecular and cellular approaches uncover the distinct mechanisms underlying these defects. First, genome-level microarray expression profiling in combination with an effective filtering criteria identify key lens genes among the mis-regulated targets in *Celf1* lens-specific conditional knockout mice. Using Celf1-RNA-immunoprecipitation (RIP) and cross-linking immunoprecipitation (CLIP) assays on lens tissue, we identify the fiber differentiation-associated nuclease Dnase2b and the ubiquitously important cell cycle regulator p27^Kip1^ (Cdkn1b) mRNAs among the endogenous direct targets of Celf1 in wild-type lens. Interestingly, Dnase2b is significantly down-regulated on the transcript level, while p27^Kip1^ is abnormally elevated on the protein level in *Celf1* deficient lenses. Further, another cyclin-dependent kinase inhibitor, p21^Cip1^ (Cdkn1a), is also up-regulated on both the mRNA and protein levels in *Celf1* deficient lenses. Using reporter analysis in lens cells, we show that Celf1 inhibits p27^Kip1^ translation via its 5’UTR, which harbors a GU-rich Celf1-binding motif, whereas Celf1 over-expression causes an elevation of Dnase2b 3’UTR fused-reporter transcripts. These findings are physiologically relevant because in differentiating lens fiber cells cyclin-dependent kinase inhibitors need to be down-regulated so that Cdk1 (Cyclin-dependent kinase 1) can be activated for phosphorylation of lamin A/C, resulting in nuclear envelope breakdown [14], which in combination with Dnase2b [15] is necessary for nuclear degradation and lens transparency. In agreement, we find lamin A/C phosphorylation defects in *Celf1* deficient mouse lenses. Thus, our data shows that Celf1 is necessary for high levels of the DNA-degrading enzyme Dnase2b, as well as its access to fiber cell nuclear DNA (by controlling p27^Kip1^, p21^Cip1^, and lamin A/C phosphorylation) to facilitate degradation of nuclei. Furthermore, we uncover yet other new Celf1 targets that provide an explanation for lens fiber cell morphology defects in *Celf1* deficient mice. We find the cell membrane organization/stability protein Sptb (Spnb1, Beta-spectrin) splice isoforms to be mis-regulated in *Celf1* deficient mice lenses suggesting that Celf1 controls alternative splicing in the lens. Additionally, we find that Celf1 directly binds to the mRNA of the F-actin-binding protein Actn2, which is down-regulated in *Celf1* deficient lens, thus explaining the defects in their fiber cell morphology. These findings provide a new RBP-associated molecular mechanism for the pathology of the eye disease cataract, while demonstrating that core regulators of cell division, as well as those involved in determining cell morphology, can be recruited by RBPs to coordinate cellular differentiation.

## Results

### Celf1 expression is conserved in vertebrate lens development

**Fig 1:**
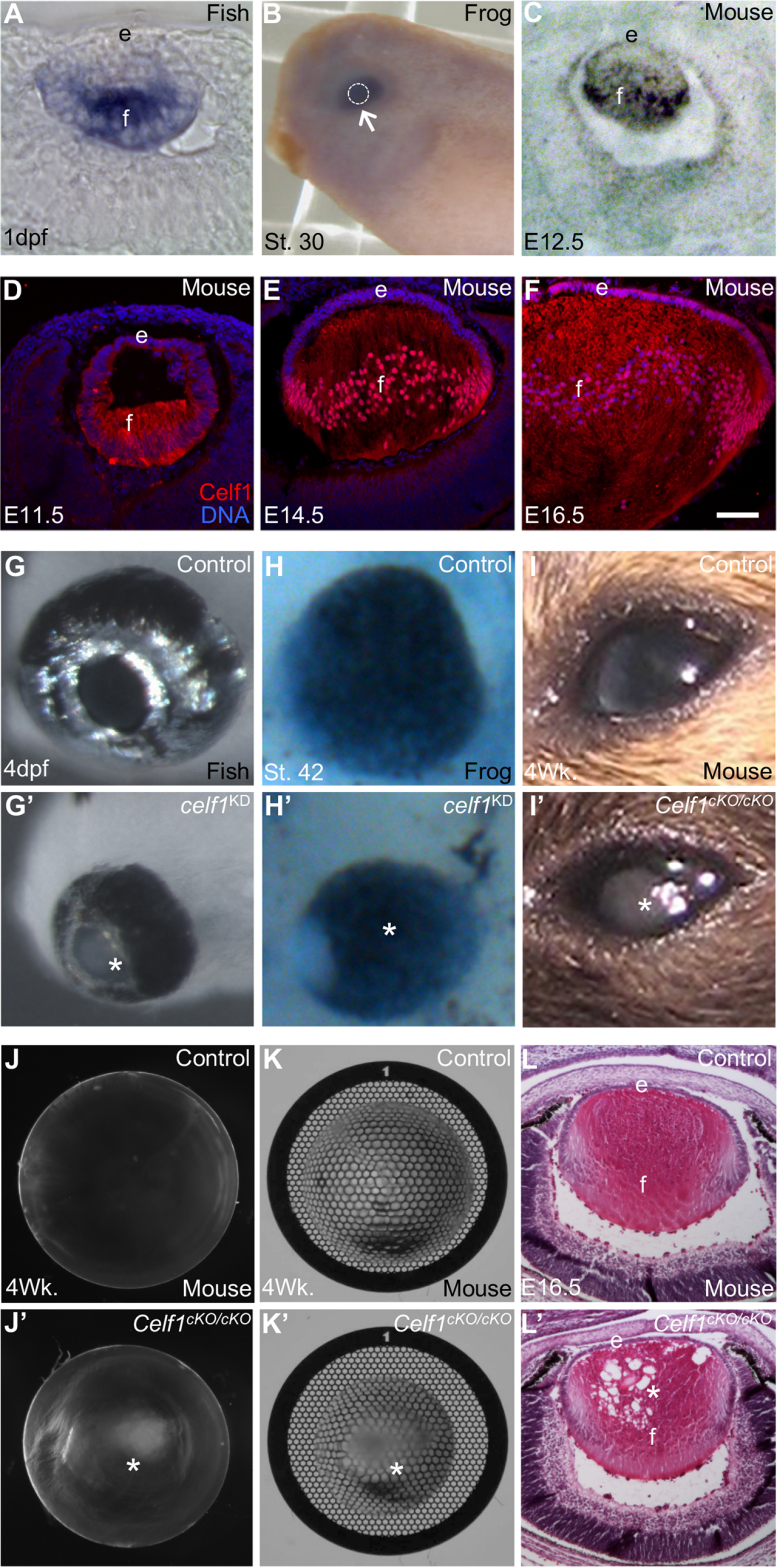
*Celf1* is required for vertebrate lens development. (**A**) In zebrafish, *celf1* transcripts are detected in the lens at 1 day post fertilization (1dpf) by *in situ* hybridization (ISH). (**B**) In *X*. *laevis*, ISH indicates strong *celf1* expression (arrow) in the embryonic St. 30 eye (arrow) and lens (indicated by broken line area). (**C**) In mouse, ISH shows strong *Celf1* transcript expression in the lens at embryonic day 12.5. (**D**) In mouse lens, Celf1 protein is expressed at (**D**) E11.5 (**E**) E14.5 and (**F**) E16.5 predominantly in fiber cells (f) and to a lower extent in epithelial cells (e). (**G**, **G’**) In zebrafish, while control eyes are normal, *celf1* knockdown (KD) results in microphthalmia and clouding of lens (asterisk) by 4dpf. (**H, H’**) In *X*. *laevis*, compared to control, *celf1* KD results in microphthalmia. (**I, I’**) In mouse, compared to control, *Celf1*^*cKO/cKO*^ lens exhibits severe cataract (asterisk). (**J**-**K’**) Compared to control, refraction errors (asterisk) are observed in *Celf1*^*cKO/cKO*^ lens under dark-field and light-field microscopy. (**L**, **L’**) At E16.5 stage, the mouse *Celf1*^*cKO/cKO*^ lens exhibits abnormal spaces (asterisk) in the fiber cell region. Scale bar in F is 75 μm.

We used an eye gene discovery tool *iSyTE*, which has previously led to the characterization of several cataract-linked genes [16–18], to identify Celf1 as a potential new regulator of lens development. The *iSyTE* approach is based on meta-analyzed microarray gene expression data on wild type mouse lens development [17,19]. It uses a strategy termed “Whole embryonic body tissue (WB) *in silico* subtraction” which is a comparative analysis of lens data with mouse WB, which allows identification of candidate genes with high lens-enriched expression. Analysis of the mouse genome using *iSyTE* tracks identified Celf1 with a high lens-enriched expression score–among top 2% (S1*A* and S1*B* Fig). The spatio-temporal expression pattern of *Celf1* in embryonic lens development is conserved in fish (Zebrafish, *Danio rerio*), amphibians (Frog, *Xenopus laevis*) and mammals (Mouse, *Mus musculus*) (Fig 1*A*–1*C*, S1*C*–S1*F’* Fig). In zebrafish, celf1 mRNA is expressed early in lens development at 1 day post fertilization (dpf) (Fig 1*A*) and elevates by 4dpf in the posterior lens where fiber cells differentiate (S1*C*–S1*E*’ Fig), which is also validated by transgenic enhancer analyses (*Tg-1*.*2celf1*:*nucGFP*) (S2*A*–*S2I”* Fig). In *X*. *laevis*, celf1 mRNA (S2*F* and S2*F’* Fig) and protein (S2*J* and S2*J’* Fig) expression in the lens is detected at early tail-bud stage (St. 23) and increases at late tail-bud stage (St. 27–30) (Fig 1*B*). In mouse, Celf1 mRNA and protein expression is high in lens fiber cells and low in the lens epithelium (Fig 1*C*–1*F*). While Celf1 protein stays high in fiber cells, its expression progressively increases in the epithelium (S2M–S2P”‘ Fig). Celf1 expression in the mouse lens is also confirmed by knock-in reporter analysis (S2*Q* and S2*Q’* Fig). Together, these data indicate that Celf1 expression is conserved in vertebrate eye development.

### Celf1 deficiency causes eye defects in fish, frog and mouse

To investigate Celf1 function in mammalian lens development, we generated a new *Celf1* conditional knockout mouse line (*Celf1*^*cKO/cKO*^) using a lens-expressed *Cre*-driver line (*Pax6GFPCre*) [20]. To minimize inefficient conditional gene deletion, we generated compound conditional knockout mice (*Celf1*^*cKO/lacZKI*^) that carried one conditional knockout allele and one germline knockout allele (S3*A*–S3*F* Fig). In *Celf1*^*cKO/lacZKI*^ mice, low level of Celf1 protein was observed at E14.5 (S3E Fig). We additionally characterized *Celf1* germline knockout/knock-in mice (*Celf1*^*lacZKI/lacZKI*^) that were previously generated [11]. In zebrafish and *X*. *laevis*, morpholinos were used to generate *celf1* knockdown (*celf1*^*KD*^) morphants (S4*A*–S4*E* Fig). *Celf1* deficiency in all systems resulted in eye defects, suggesting an evolutionarily conserved requirement for Celf1 in eye development (Fig 1*G*–1*I’*). In zebrafish, *celf1* morphants exhibit lens defects and cataracts at 4dpf (Fig 1*G* and 1*G’*; S5*A* Fig) while in *X*. *laevis*, majority of *celf1* morphants (55.7%, *n* = 136) exhibit microphthalmia (small eye) (Fig 1*H* and 1*H’*) and a minority (27%, *n* = 66) exhibit a skewed eye defect (defined as distorted eyes with abnormal appearance of retina and lens tissue) (S5*B*–S5*D* Fig). In mouse, all three *Celf1* homozygous deletion mouse genotypes–*Celf1*^*cKO/cKO*^, *Celf1*^*cKO/lacZKI*^ and *Celf1*^*lacZKI/lacZKI*^–exhibit severe lens defects including cataracts at early postnatal (P) stages (Fig 1*I*–1*K’*; S6*A* and S6*B’* Fig). In *Celf1*^*cKO/lacZKI*^ mice, lens fiber cells exhibit abnormal spaces at E16.5 (Fig 1*L* and 1*L’*), suggesting that perturbation of the fiber differentiation program causes cellular morphological defects that underlie the cataract phenotype. In sum, while the cataracts were obvious in zebrafish *celf1* morphants and mouse *Celf1* knockouts, and the frog *celf1* morphants had small lenses, obvious cataracts were not detected in frogs. In addition to ocular defects, frog *celf1* morphants exhibit somite segmentation defects (S7 Fig), similar to earlier reports [21]. Collectively, these findings demonstrate that Celf1 is necessary for lens development in vertebrates.

### Molecular insights into Celf1 deficiency-mediated lens defects

**Fig 2:**
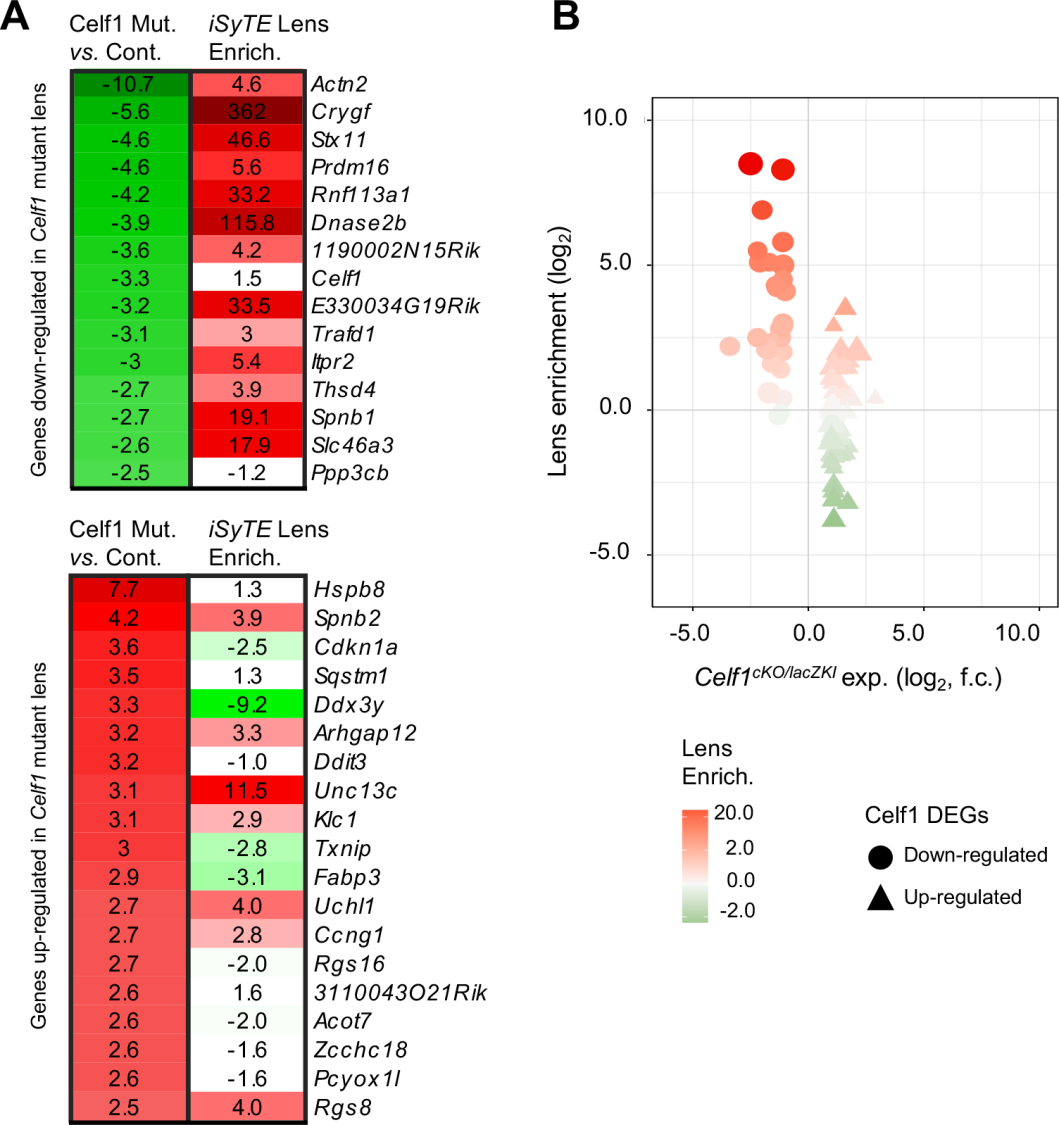
*Celf1*^*cKO/lacZKI*^ mouse exhibits mis-expression of key lens genes. (**A**) Microarray heat-maps representing genes mis-regulated in *Celf1*^*cKO/lacZKI*^ lenses compared to control (left column, ±2.5 fold-change, *p*<0.05, total 34 genes, indicated by heatmap color gradients (left columns: green, down in *Celf1*^*cKO/lacZKI*^; red, up in *Celf1*^*cKO/lacZKI*^) and their respective enrichment in normal lens compared to whole-embryonic tissue as per *iSyTE* (right columns, lens-enrichment in fold-change indicated by red color intensity). (**B**) Differentially expressed genes (DEGs) in *Celf1*^*cKO/lacZKI*^ lenses are plotted on the X-axis as down-regulated (circles) and up-regulated genes (triangles). On the Y-axis, DEGs are separated based on their lens-enrichment. Red and green color gradients represent high and low lens-enrichment, respectively. Genes down-regulated in *Celf1*^*cKO/lacZKI*^ lenses are predominantly highly-lens enriched, while those up-regulated do not exhibit this trend.

To understand the molecular mechanism underlying Celf1 function in mouse lens development, we first performed genome-level expression profiling in newborn *Celf1*^*cKO/lacZKI*^ lenses, which identified several differentially expressed gene candidates (DEGs) (Fig 2*A*). Further, analysis of the *Celf1*^*cKO/lacZKI*^ lens DEGs by comparing them to normal mouse lens gene expression data in *iSyTE* shows that majority of the genes down-regulated in *Celf1*^*cKO/lacZKI*^ lenses exhibit highly enriched expression in lens development, while up-regulated genes had no such pattern (Fig 2*B*)–indicating that Celf1 is necessary for expression of genes associated with differentiating fiber cells. To identify high-priority targets among *Celf1*^*cKO/lacZKI*^ lens DEGs, we next applied an effective filtering criteria that in the past has successfully pointed to key genes that explained the lens phenotype in other gene knockout mice [18,22–24]. These analyses identify known–as well as new–candidate genes involved in different aspects of fiber cell differentiation, in turn offering avenues for detailed investigation (see below) for explaining the cataract pathology observed in Celf1 deficient lenses.

### Celf1 deficiency affects Dnase2b mRNA levels and lamin A/C phosphorylation, causing fiber nuclear degradation defects

**Fig 3:**
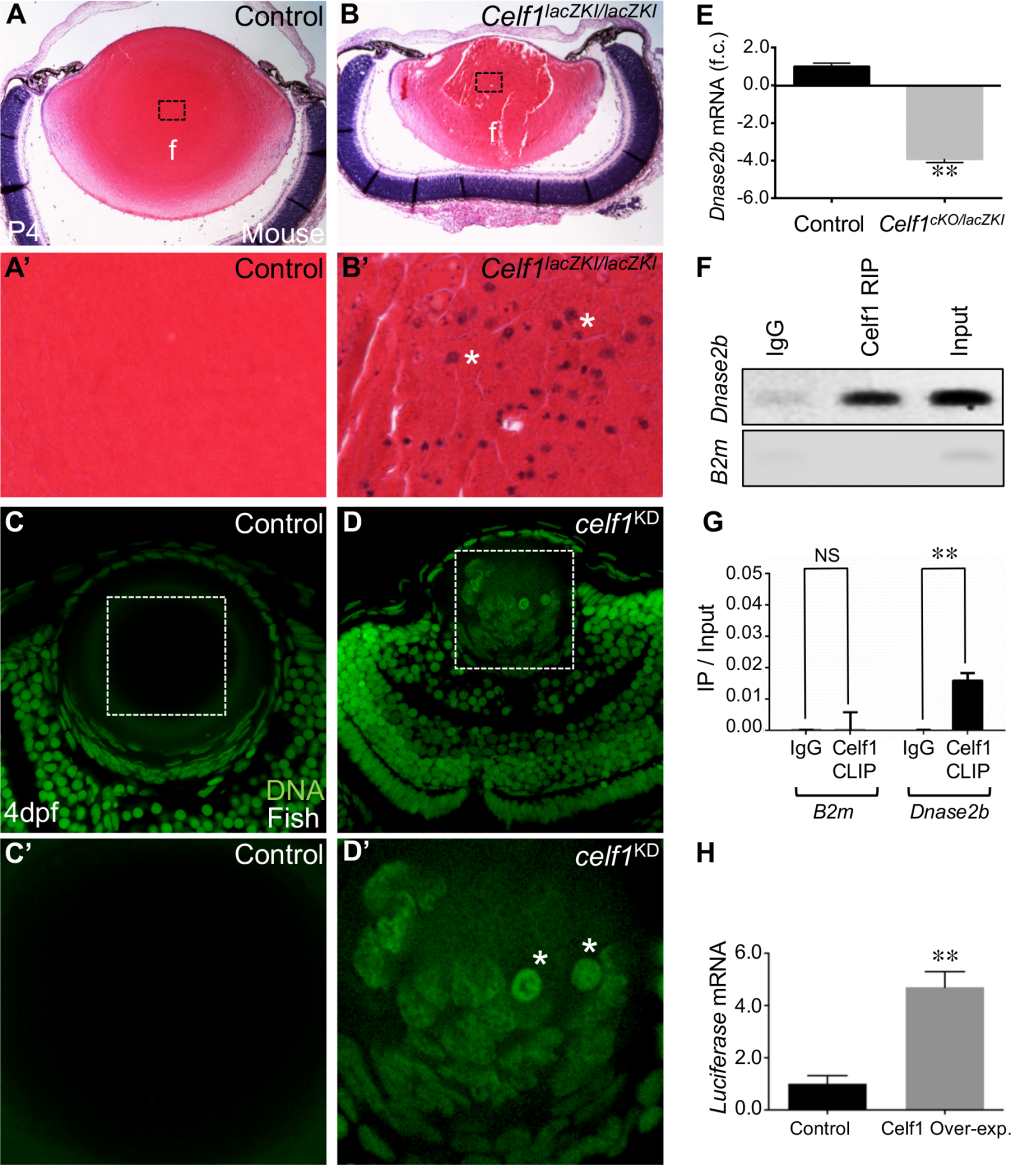
*Celf1* deficiency in mouse and fish causes fiber cell nuclear degradation defects. (**A, B**) Histological analysis of control and *Celf1*^*lacZKI/lacZKI*^ mouse lenses at post natal day 4 (P4) stage shows abnormal presence of nuclei in centrally located fiber cells only in *Celf1*^*lacZKI/lacZKI*^ mice. (**A’, B’**) High-magnification of the dotted-line area in A, B. Asterisk denote abnormally retained nuclei. (**C, D**) In zebrafish, compared to control, *celf1*^*KD*^ lens exhibit abnormal presence of nuclei in the central fiber cell region. (**C’, D’**) High-magnification of the dotted-line area in E, F. Asterisk denote abnormally retained nuclei. (**E**) RT-qPCR analysis confirms significant *Dnase2b* down-regulation in *Celf1*^*cKO/lacZKI*^ lenses compared to control. (**F**) RNA immunoprecipitation (RIP) and (**G**) cross-linked RNA immunoprecipitation (CLIP) shows *Dnase2b* to be enriched in Celf1-pulldown in wild-type mouse lens. (**H**) Celf1 over-expression in NIH3T3 cells, which carry Dnase2b 3’UTR downstream of a luciferase reporter, results in significant increase of luciferase mRNA. Abbr.: f.c., fold-change; NS, not significant. Asterisks in E, G, H indicate a *p*-value < 0.005.

**Fig 4:**
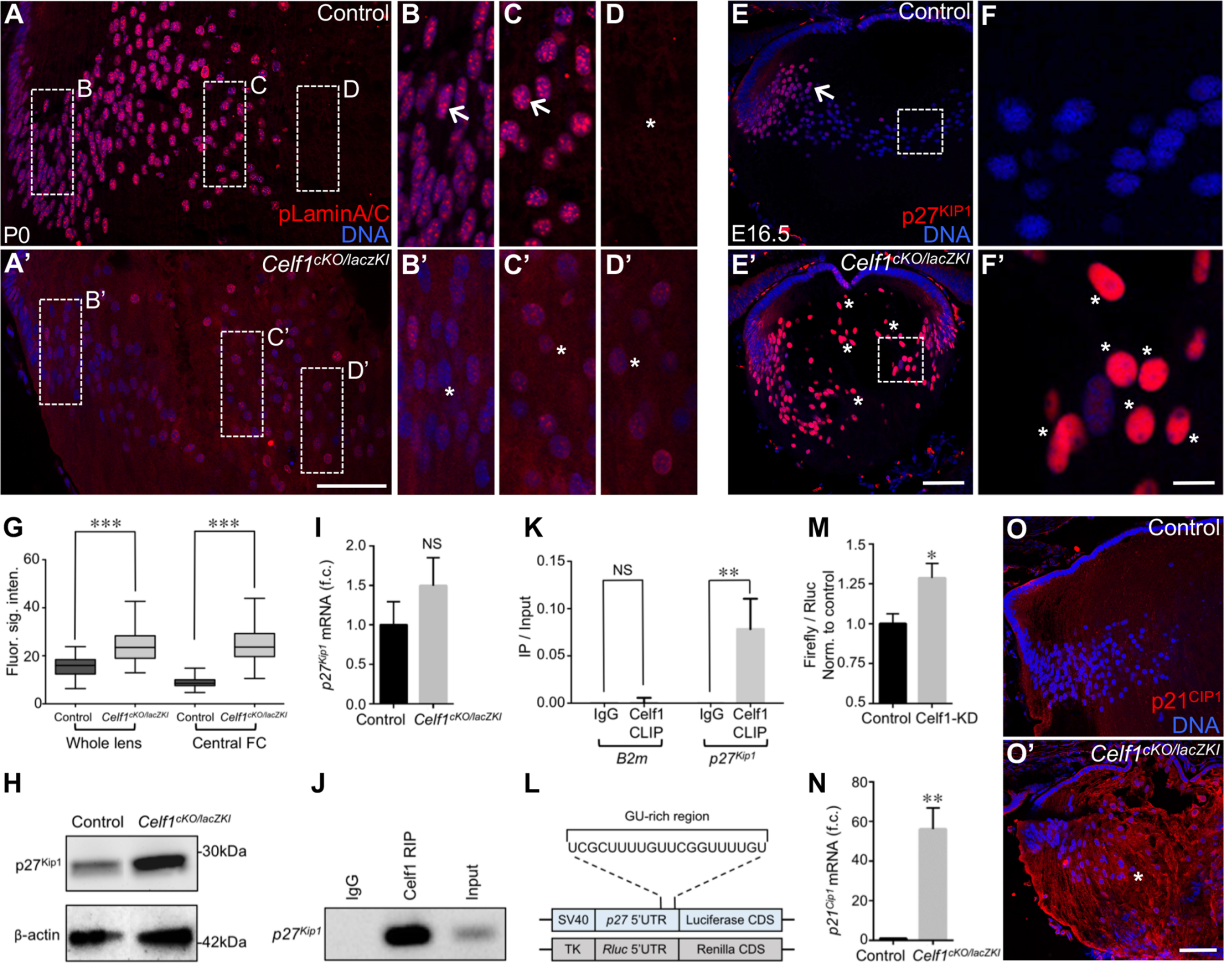
Celf1-mediated post-transcriptional control of mitotic machinery components facilitates lens fiber cell nuclear degradation. (**A, A’**) Compared to control lens, phosphorylation of Lamin A/C (pLamin A/C) is reduced in *Celf1*^*cKO/lacZKI*^ lens. Dotted-line areas of the fiber cell regions in control (**B**-**D**) and *Celf1*^*cKO/lacZKI*^ mice (**B’**-**D’**) lenses are shown at high-magnification. Arrows in B, C indicate examples of high pLamin A/C expressing nuclei, while no nuclei are observed in D as this area represents a normal nuclear-free zone in the control lens. Asterisks in B’-D’ indicate reduced signals of pLamin A/C in *Celf1*^*cKO/lacZKI*^ lens fiber cell nuclei. Note the presence of nuclei in D’ in the centrally located fiber cells due to the nuclear degradation defects in the *Celf1*^*cKO/lacZKI*^ lens. (**E**, **E’**) Unlike in the control lens where p27^Kip1^ protein is restricted to cells of the transition zone and cortical fiber cells (arrow), high levels of p27^Kip1^ protein are detected in the entire fiber cell compartment (asterisks) including the central region in *Celf1*^*cKO/lacZKI*^ lens. (**F, F’**) High-magnification of dotted-line areas in E and F. Asterisks indicate elevated signals of p27^Kip1^ protein. (**G**) Quantification of the p27^Kip1^ immunofluorescence signals from control and *Celf1*^*cKO/lacZKI*^ lenses shows significantly increased p27^Kip1^ protein levels in *Celf1*^*cKO/lacZKI*^ lenses. (**H**) Western blot analysis shows increased levels of p27^Kip1^ protein in *Celf1*^*cKO/lacZKI*^ lens compared to control. (**I**) Compared to control, p27^Kip1^ mRNA levels are not significantly altered in *Celf1*^*cKO/lacZKI*^ lens. (**J, K**) RIP and CLIP assays, respectively, identify p27^Kip1^ mRNA to be highly enriched in Celf1-pulldown in wild-type mouse lens. (**L**) A potential Celf1 binding GU-rich region in present in the mouse p27^Kip1^ 5’ UTR. (**M**) Activity of firefly luciferase fused downstream of p27^Kip1^ 5’ UTR is significantly elevated in Celf1-knockdown (Celf1-KD) mouse lens cell line compared to control (firefly luciferase normalized to *Renilla* luciferase (*Rluc*)). (**N**-**O’**) Compared to control, p21^Cip1^ mRNA and protein is abnormally elevated in *Celf1*^*cKO/lacZKI*^ lens. Asterisk in O’ indicate high expression of p21^Cip1^ protein in the *Celf1*^*cKO/lacZKI*^ lens. Abbr.: e, epithelial cells; f, fiber cells; tz, transition zone; NS, not significant. Scale bar in A’, E’, O’ is 75 μm and in D’ and F’ is 12 μm. Asterisks in G, K, M and N indicate a *p*-value < 0.0005, 0.005, 0.05 and 0.005, respectively.

Interestingly, among the *Celf1*^*cKO/lacZKI*^ lens high-priority DEGs, *Dnase2b* –a lysosomal enzyme that is highly enriched in normal lens development [15,25], is down-regulated in *Celf1*^*cKO/lacZKI*^ lenses (Fig 2*A*). This is of significance because Dnase2b is necessary for fiber cell nuclear degradation during terminal differentiation, and mice deficient for this gene exhibit cataracts [15,26]. We therefore examined the consequence of *Dnase2b* down-regulation in *Celf1*^*lacZKI/lacZKI*^ early postnatal lens and detected the abnormal retention of nuclei in centrally located fiber cells (Fig 3*A*–3*B’* and S8 Fig). This defect is observed in all three types of *Celf1* knockout mouse lenses, even at later stages, indicating that the nuclear degradation pathway is not simply delayed but is fundamentally defective (S9*A*–S9*F”* Fig). Strikingly, zebrafish *celf1* morphant lenses also exhibit defective fiber cell nuclear degradation, suggesting a functional conservation of Celf1 in developing fish and mammalian lenses (Fig 3*C*–3*D’*; S9*G*–S9*H’* Fig). We next examined the microarray data for altered expression of other factors involved in nuclear degradation, but did not uncover significant changes in such factors in the *Celf1*^*cKO/lacZKI*^ lens data. While this analysis does not rule out translational level changes in these factors, it reinforced Dnase2b as a key candidate for further investigation in *Celf1*^*cKO/lacZKI*^ lens. Dnase2b mRNA down-regulation in *Celf1*^*cKO/lacZKI*^ lenses is confirmed by RT-qPCR (Fig 3*E*). Further insight into Celf1-mediated Dnase2b control is gained from RNA-immunoprecipitation (RIP) and cross-linking immunoprecipitation (CLIP) assays that demonstrate an enrichment of Dnase2b mRNA in the Celf1 antibody pulldown on normal mouse lens (stage P15) (Fig 3*F* and 3*G*). These findings identify Dnase2b as a direct target of Celf1 and lead to the hypothesis that this RBP-RNA interaction is necessary for elevated Dnase2b transcript levels. Indeed, Celf1 overexpression in NIH3T3 cells that carry a luciferase reporter-Dnase2b 3’ UTR fusion construct result in elevated levels of luciferase reporter transcripts (Fig 3*H*; S10*A* and S10*B* Fig). Together, these data indicate that Celf1 functions to control Dnase2b mRNA levels through interactions with its 3’UTR. In normal fiber differentiation, in addition to optimal levels of Dnase2b, phosphorylation of nuclear envelope proteins lamins A and C is also necessary for the breakdown of the nuclear membrane so that Dnase2b can gain access to fiber nuclear DNA [14]. In *Celf1*^*cKO/lacZKI*^ lenses, fiber cell nuclei exhibit reduced phosphorylation of lamin A/C (Fig 4*A*–4*D’*; S11*A–S*11*F* Fig). Interestingly, there is variation in the levels of phospho-lamin in the fiber cell nuclei of *Celf1*^*cKO/lacZKI*^ lens, which may reflect the presence of residual Celf1 protein in a subset of fiber cells in the *Celf1*^*cKO/lacZKI*^ lens. Together, these findings indicate that defective phosphorylation of nuclear lamins and reduced Dnase2b levels together contribute to the fiber cell nuclear degradation defects in *Celf1*^*cKO/lacZKI*^ lenses.

### Celf1 re-wires mitotic machinery components to control fiber cell nuclear envelope breakdown

We next investigated the molecular basis of the lamin A/C phosphorylation defect in *Celf1*^*cKO/lacZKI*^ mice. It is established that expression of the cyclin-dependent kinase (Cdk) inhibitor protein p27^Kip1^ (Cdkn1b) gets elevated in epithelial cells located in the lens transition zone, where it functions in coordinating their cell cycle exit and commitment to fiber differentiation [27]. In later stages of fiber differentiation, a sharp reduction of p27^Kip1^ protein is necessary for activation of the cyclin-dependent kinase Cdk1, which in turn phosphorylates nuclear lamin A/C to initiate nuclear envelope disassembly [14,28]. However, the mechanism underlying the sharp reduction of p27^Kip1^ is not understood [29].

We find that p27^Kip1^ protein levels are abnormally high in *Celf1*^*cKO/lacZKI*^ lens fiber cells without an accompanying significant increase in its mRNA (Fig 4*E*–4*I*; S12*A* Fig). This lack of transcript-level changes of p27^Kip1^ in the *Celf1*^*cKO/lacZKI*^ lens suggests that this factor may be regulated at the post-transcriptional level in the lens. In the *Celf1*^*cKO/lacZKI*^ lens, at E16.5, a few nuclei of the lens epithelium exhibited elevated p27^Kip1^ protein expression compared to control, but this subtle defect was not observed by stage P0 (S12*B* Fig). However, the *Celf1*^*cKO/lacZKI*^ lens fiber cells continued to express high p27^Kip1^ protein levels at P0 (S12*C* and S12*D* Fig). Together, these data suggest that Celf1 normally functions to post-transcriptionally inhibit p27^Kip1^ protein expression in late stages of fiber differentiation. In support of this, p27^Kip1^ transcripts are enriched in Celf1-RIP and CLIP pulldown assays (Fig 4*J* and 4*K*), suggesting that Celf1 directly associates with p27^Kip1^ mRNA in the developing mouse lens. Several studies have shown that Celf1 binds to GU-rich motifs in target RNAs. These are based on SELEX (systematic evolution of ligands by exponential enrichment) assays [30] and by identifying conserved motifs within various Celf1-controlled transcripts [31–33]. Further, the structural bases for the strong preference of Celf1 for GU-rich element binding has also been demonstrated by biophysical approaches [34]. Accordingly, the p27^Kip1^ mRNA 5’UTR has a GU-rich element that represents a potential Celf1 binding region (Fig 4*L*). Therefore, to test the hypothesis that Celf1 directly represses p27^Kip1^ translation, we generated stable shRNA-mediated *Celf1*-knockdown (*Celf1*-KD) (S13 Fig) in a well characterized mouse lens-derived cell line 21EM15 [35] and transfected it with p27^Kip1^ 5’UTR fused to a luciferase ORF reporter construct driven by the SV40 promoter. *Celf1*-KD cells had significantly elevated luciferase reporter levels compared to control (Fig 4*M*), indicating a release of p27^Kip1^ translational inhibition upon Celf1 reduction. Together, these data suggest that Celf1 represses the translation of p27^Kip1^ in differentiating fiber cells through direct interaction with its 5’UTR. Interestingly, Celf1-mediated translational repression of p27^Kip1^, potentially through interference of an internal ribosome entry site (IRES) in its 5’UTR, is observed in cultured breast cancer cells [36], but has never before been described in developing tissue. Further, similar to p27^Kip1^, the Cdk-inhibitor p21^Cip1^ (Cdkn1a) functions to regulate the cell cycle by inducing growth arrest [37]. While in normal lens development, p21^Cip1^ is repressed [27], we find both p21^Cip1^ mRNA (among the high-priority DEGs) and protein to be abnormally elevated in *Celf1*^*cKO/lacZKI*^ lens (Fig 2A, Fig 4*N*–4*O’*). This is of significance given that elevated levels of p21^Cip1^ has been linked to cataract [38]. We also performed Celf1 CLIP-RTqPCR on mouse lenses for p21^Cip1^, but did not detect p21^Cip1^ transcripts to be enriched in the Celf1 pulldowns. This may be because the lens does not normally express p21^Cip1^ transcripts [27]. However, the direct interaction of Celf1 with p21^Cip1^ has been investigated in HeLa cells that have abundant expression of p21^Cip1^ mRNA [39], and in this dataset CLIP identifies p21^Cip1^ as a direct binding target of Celf1. Together, our findings indicate that Celf1 is necessary to negatively control the expression of the cell cycle regulators p27^Kip1^ and p21^Cip1^ and the phosphorylation status of lamin A/C to orchestrate fiber cell nuclear envelope breakdown in lens development.

### Celf1 deficiency affects Actn2 and Sptb and causes abnormal fiber cell morphology

**Fig 5:**
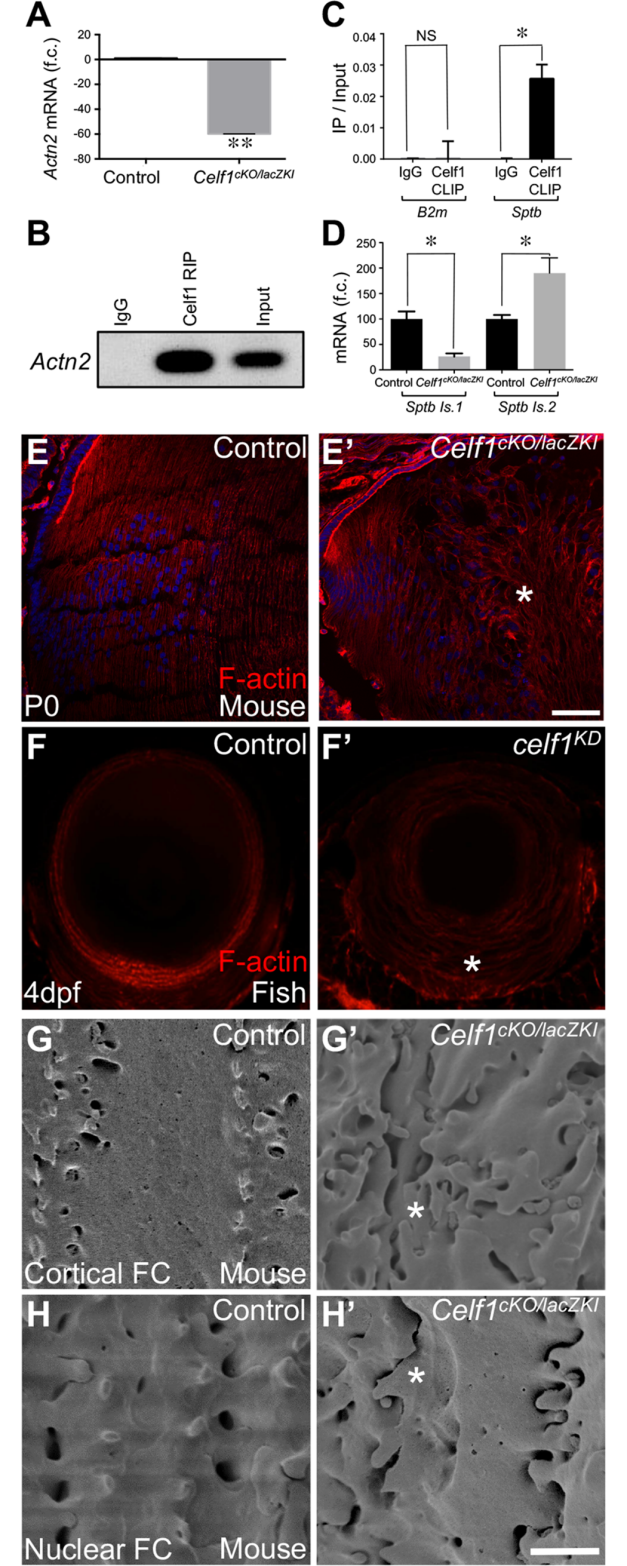
*Celf1* deficiency in mouse and fish causes defects in fiber cell morphology. (**A**) RT-qPCR analysis confirms significant *Actn2* down-regulation in *Celf1*^*cKO/lacZKI*^ lenses compared to control. (**B**) RNA immunoprecipitation (RIP) identifies *Actn2* as an enriched transcript in Celf1-pulldown in P15 wild-type mouse lens. (**C**) Cross-linked RNA immunoprecipitation (CLIP) shows *Sptb* transcripts to be enriched in Celf1-pulldown in wild-type mouse lens. (**D**) RT-qPCR analysis shows that the high-abundant *Sptb* isoform (isoform 1 (ENSMUST00000021458)) is reduced, while the low-abundant *Sptb* isoform (isoform 2 (ENSMUST00000166101)) is abnormally elevated in *Celf1*^*cKO/lacZKI*^ lenses. (**E, E’**) In mouse, phalloidin staining of lens tissue sections (stage P0) shows uniform F-actin deposition along the fiber cell margins in control, while *Celf1*^*cKO/lacZKI*^ lenses exhibit abnormal pattern of F-actin (asterisk). (**F, F’**) In zebrafish, while control lens exhibits normal F-actin deposition, *celf1*^*KD*^ lens (stage 4dpf) exhibits abnormal F-actin pattern (asterisk) in fiber cells. (**G**-**H’**) In mouse, scanning electron microscopy analysis of cortical and nuclear fiber cells shows disrupted cell organization (asterisk) in *Celf1*^*cKO/lacZKI*^ lenses (stage P15). Scale bar in D’ is 75 μm and G’ is 2.5μM.

The severe fiber cell defects observed in *Celf1*^*cKO/lacZKI*^ mice (Fig 1*L* and 1*L’*) suggest its potential function in controlling fiber cell morphology/organization. In agreement with this, among the *Celf1*^*cKO/lacZKI*^ lens DEGs, transcripts for *Actn2* (α-actinin 2; F-actin crosslinking protein) and *Sptb* (also known as *Spnb1*; β-spectrin; protein required for cell membrane organization/stability) are among the high-priority candidates that are significantly downregulated (Fig 2*A*). The reduced *Actn2* transcript levels were confirmed by RT-qPCR (Fig 5*A*). Actn2 is a spectrin family protein that contains a conserved actin-binding domain to facilitate crosslinking of actin [40]. Interestingly, *Actn2* knockdown in zebrafish results in lens defects and microphthalmia [41]. Further, *iSyTE* analysis identifies *Actn2* as a lens-enriched gene in development (Fig 2A). These findings offer a hypothesis that the abnormalities in fiber cell morphology in *Celf1*^*cKO/lacZKI*^ lenses may be reflective of Actn2 downregulation-mediated cytoskeletal defects. Therefore, we next tested potential interactions between Celf1 protein and *Actn2* mRNA by performing RNA immunoprecipitation (RIP) on normal mouse lenses (stage P15). RIP analysis identified *Actn2* as an enriched transcript in Celf1-pull down lysates but not in the IgG control (Fig 5B), suggesting that Celf1 directly interacts with *Actn2* mRNA.

Next, we investigated Sptb (β-spectrin) expression in more detail in the *Celf1*^*cKO/lacZKI*^ lens. Spectrins (classified as α- and β-spectrins) are membrane skeletal proteins that line the cell membrane mediating its stability by positioning transmembrane proteins and thereby controlling cell shape [42]. Further, spectrins crosslink with actin filaments, which is important for fiber cell packing [43,44]. We performed CLIP-RTqPCR on mouse postnatal day 13 lens and identified *Sptb* transcripts in the Celf1 pulldown, suggesting that Celf1 directly regulates *Sptb* transcripts (Fig 5C). We identified two differentially expressed Sptb mRNA splice isoforms in normal lenses, the high-expressed Sptb isoform 1 (ENSMUST00000021458, contains 35 exons and codes for a splice isoform with a longer C-terminus region) and the low-expressed Sptb isoform 2 (ENSMUST00000166101, contains 31 exons and codes for a splice isoform with a shorter C-terminus region) (S14 Fig). Using RT-qPCR, we find the abundance of these isoforms to be affected in the *Celf1*^*cKO/lacZKI*^ lenses, with Sptb isoform 1 being reduced and Sptb isoform 2 being elevated (Fig 5D). Spbt isoform 1 contains the pleckstrin domain in its extended C-terminal region, which is involved in membrane interactions, and based on its expression, is the predominant isoform in the lens. Thus, the abnormal levels of the two Sptb isoforms, along with reduced Actn2, may contribute toward the fiber cell morphology defects in *Celf1*^*cKO/lacZKI*^ lenses.

To determine the impact of these mis-regulated cyto- and membrane-skeletal factors, we investigated F-actin pattern in *Celf1*^*cKO/lacZKI*^ and control lenses in mouse and fish. In mouse, phalloidin staining of lens sections shows that while F-actin levels are not altered significantly, it appears abnormal, likely secondary due to cytoskeletal defects or the gross cellular disorganization in *Celf1*^*cKO/lacZKI*^ lenses (Fig 5*E* and 5*E’*). Similarly, in zebrafish, compared to control, F-actin appears abnormal in *celf1* knockdown lenses (Fig 5*F* and 5*F’*). These findings demonstrate a critical function for Celf1 in maintaining fiber cell cytoskeletal structure in lens development. Further, in mouse, scanning electron microscopy (SEM) was performed to analyze cortical fiber cells (located in the lens outer cortex) and nuclear fiber cells (located near the core of lens) at stage P15. In control lenses, both cortical and nuclear fiber cells are arranged in a discrete parallel arrangement that interlink with the neighboring fiber cells through membrane protrusions (Fig 5*G* and 5*H*). In contrast, both cortical and nuclear fiber cell organization is severely abnormal in *Celf1*^*cKO/lacZKI*^ lens, exhibiting irregular arrangement of membrane protrusions and inter-digitations between neighboring cells (Fig 5*G’* and 5*H’*). Collectively these data suggest that Celf1 deficiency causes severe alterations in mRNA levels or specific isoform abundance of key genes *Actn2* and *Sptb* that results in abnormal fiber cell morphology.

## Discussion

While signaling, transcriptional, epigenetic and non-coding RNA-mediated gene expression control is well characterized, the importance of RBP-driven post-transcriptional control in vertebrate organogenesis is not well defined, barring a few exceptions [13,45]. Further, for the past 50 years, the ocular lens has been studied as an extreme example of cell type-specific gene expression, largely focusing on the transcriptional control of crystallin genes in cells that eventually degrade their nuclei and other organelles to achieve transparency. Here, we demonstrate that a conserved RBP is necessary for the dynamic spatio-temporal control over the lens fiber cell proteome, and its deficiency in different vertebrates results in cataract. Thus, the long-standing dogma that transcription is the predominant factor in regulating lens fiber differentiation is refuted by these findings that highlight distinct post-transcriptional roles of Celf1 in the lens. Significantly, these data reveal a new RBP-based mechanistic layer of control–involving mRNA translation, stability or alternative splicing–over p27^Kip1^, Actn2, Dnase2b and Sptb expression during lens development.

These data establish Celf1 as an important regulator in vertebrate lens development, while shedding new light on the regulation of known–as well as several new–target genes. For example, (1) while it was known that germline deletion of Dnase2b in mouse caused nuclear degradation defects and cataract [15], the mechanism of how Dnase2b was expressed at high levels was not understood; (2) Similarly, it was shown that down-regulation of p27^Kip1^ is necessary for nuclear degradation in differentiating fiber cells [28], but how this p27^Kip1^ control was precisely accomplished in these cells was not understood; (3) Additionally, elevated expression of p21^Cip1^ was linked to cataract [38], but which factor negatively controlled p21^Cip1^ in the lens was not understood; (4) Alternative splicing of key genes was long suspected to be important for lens development [3], but no factor controlling this post-transcriptional control mechanism was characterized in the lens; and (5) It is long known that the characteristic cellular morphology is critical for lens transparency but the regulatory mechanism to control cytoskeletal factors, including F-actin, in these elongated cells was not well understood [44]. Data in the present manuscript establish that the RBP Celf1 mediates post-transcriptional regulation to control known (p27^Kip1^, p21^Cip1^, Dnase2b), as well as novel (Actn2, Sptb), factors thereby orchestrating fiber differentiation during lens development. Although other post-transcriptional RBPs such as Tdrd7 and Caprin2 are known to function in lens development [16,46], their gene knockout mouse models do not exhibit nuclear degradation defects that are observed in the *Celf1*^*cKO/lacZKI*^ lens. This indicates that different RBPs have distinct function in lens development.

**Fig 6:**
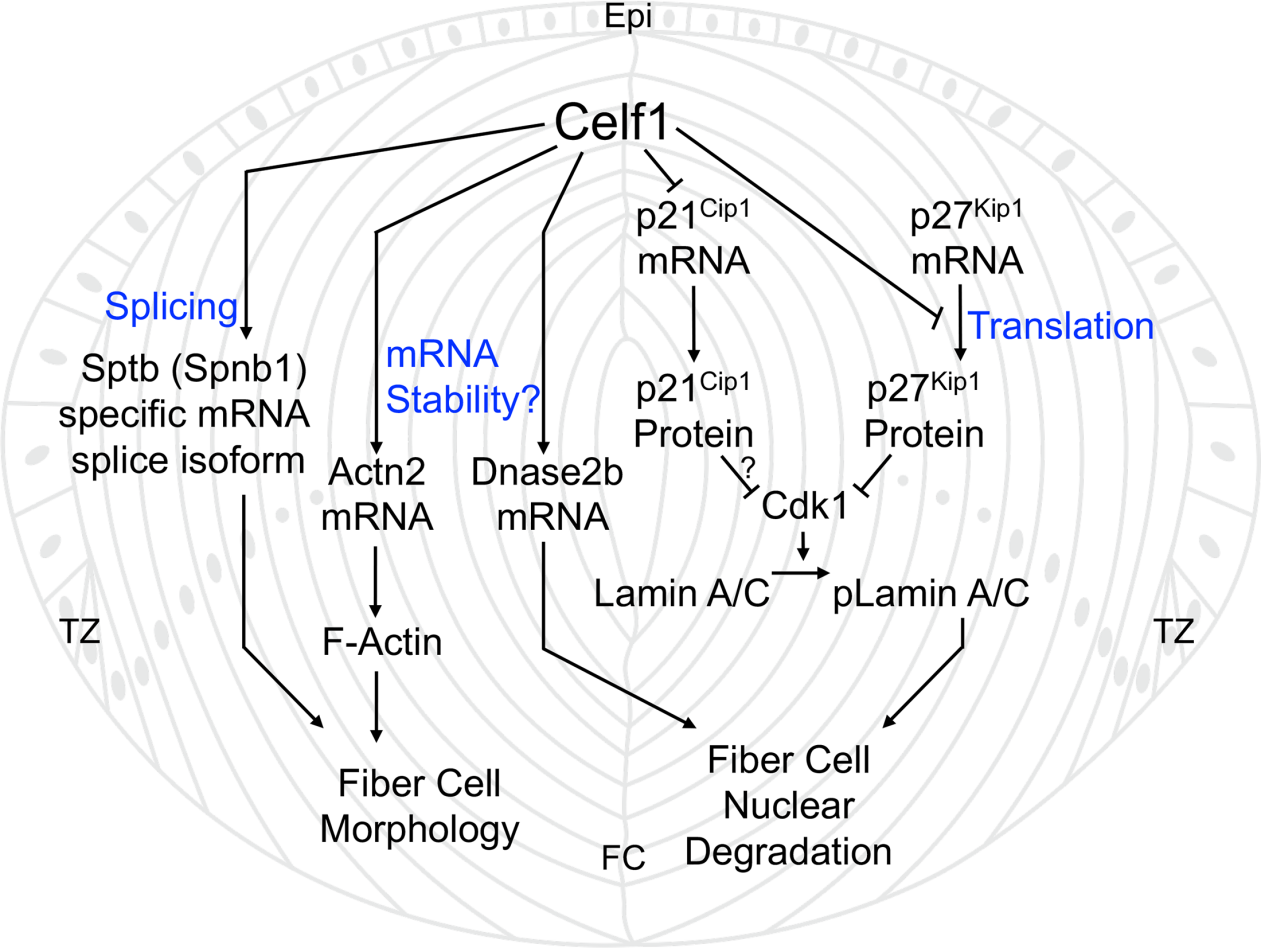
Model for Celf1-mediated post-transcriptional gene expression control in the lens. In normal lens development, Celf1 is required for nuclear degradation and proper cell morphology in fiber cell differentiation. Celf1 positively regulates the nuclease Dnase2b (being necessary for its high mRNA levels) and negatively regulates the cyclin-dependent kinase inhibitors p21^Cip1^ (being necessary for its low mRNA levels) and p27^Kip1^ (by inhibiting its translation into protein). Inhibition of p21^Cip1^ and p27^Kip1^ allows the activation of Cdk1, which phosphorylates Lamin A/C to initiate nuclear envelope breakdown in fiber cells. Thus, Celf1 controls the nuclease (Dnase2b) as well as its access to nuclear DNA, to regulate nuclear degradation in lens fiber cells. These findings show how mitotic machinery components–normally involved in nuclear envelope disassembly during cell division–are post-transcriptionally rewired by RNA-binding proteins to regulate cell differentiation in lens development. Additionally, Celf1 controls the splice isoform abundance of the membrane-organization protein Sptb (β-spectrin) and high transcript levels of the F-actin-binding protein Actn2 (α-actinin 2), to regulate fiber cell morphology. Abbr.: Epi, epithelium; TZ, transition zone; FC, fiber cells.

From these new data, and in light of previous findings [14,15,28,38], a model is defined for the molecular mechanism of Celf1 function in lens development (Fig 6). In one pathway, Celf1 positively regulates a lysosomal nuclease Dnase2b in fiber cells while also indirectly facilitating its access to nuclear DNA by negatively regulating p27^Kip1^ and p21^Cip1^. Down-regulation of these Cdk-inhibitors results in the activation of Cdk1, which phosphorylates nuclear lamins A/C to initiate fiber cell nuclear envelope breakdown, thus allowing Dnase2b access to degrade nuclear DNA. This model shows how components of the mitotic machinery–normally involved in nuclear disassembly during cell division–are rewired by RBPs to regulate cell differentiation. In separate pathways, Celf1 is necessary for appropriate levels of the F-actin crosslinking protein Actn2 (α-actinin 2), which is previously associated with lens defects [41], and also for the high abundance of the specific alternative splice isoform of Sptb, β-spectrin, which is necessary for cell membrane integrity [43]. Deficiency of Celf1 reduces these factors, culminating into fiber cell morphology defects. Together, the fiber cell nuclear degradation and morphology defects cause cataract in the *Celf1* deficient lens.

The findings described here provide new control mechanisms for universally important factors such as p27^Kip1^, p21^Cip1^, alpha-actinin, Beta-spectrin, which are involved in differentiation and morphology of a multitude of different cell types. Indeed, the Celf1-mediated p27^Kip1^ post-transcriptional control mechanism described here in the lens may serve to inform on the complexity of p27^Kip1^ regulation in other developing tissues, and the impact of its mis-regulation on cell growth and cancer, as well as on the pathobiology of other Celf1-associated defects such as myotonic dystrophy. The translational control of p27^Kip1^ has been investigated in different cell types. The 5’UTR of p27^Kip1^ has an IRES that supports cap-independent translation and other elements that allow translational control in specific phases in the cell cycle [47–49]. Importantly, the present data show for the first time that Celf1 negatively controls p27^Kip1^ translation in a developing tissue. Interestingly, the ELAV family protein HuR has been shown to inhibit p27^Kip1^ translation via the IRES in its 5’UTR in HeLa cells [50]. We have identified HuR in mouse embryonic lens [16] and it will be interesting to investigate in future studies whether this protein functions with Celf1 to cooperatively regulate p27^Kip1^ translation in the lens. Further, our new findings on Celf1-based translational regulation of p27^Kip1^, along with the findings that mutations in the 5’UTR of L-ferritin mRNA mis-regulate its translation into protein and result in hyperferritinaemia and cataract [51], together serve to highlight the importance of translational control of key factors linked to cataractogenesis. Further, while Celf1 is implicated in control of p21^Cip1^, depending on the specific cell line investigated, it has been reported to be either a positive regulator or a negative regulator of p21^Cip1^ [52,53]. Here, we show for the first time that in lens fiber cells, Celf1 negatively regulates p21^Cip1^. The findings in the present study are generally significant because RBP function in RNA processing is increasingly recognized as a critical factor for fully comprehending human disease phenotypes [54]. Together with the finding that deficiency of the post-transcriptional regulatory protein TDRD7 causes juvenile cataracts in human, mouse and chicken [16], these data highlight the importance of RBP-mediated post-transcriptional regulatory networks for precise spatiotemporal control of cellular proteomes in vertebrate organogenesis.

## Materials and methods

### Ethics statement

The University of Delaware Institutional Animal Care and Use Committee (IACUC) reviewed and approved the animal protocols (number 1226). Animal experiments were performed according to the Association for Research in Vision and Ophthalmology (ARVO) statement for the use of animals in ophthalmic and vision research.

### Generation of *Celf1* targeted deletion mouse

A new conditional knockout mouse model (*Celf1*^*cKO/cKO*^) was generated in this study wherein *Celf1* exon five is flanked by *loxP* sites (*Celf1*^*flox/flox*^) (Clinique de la Souris, Strasbourg). In addition, previously generated *Celf1* germline targeted knockout animals (referred to as *Celf1*^*lacZKI/lacZKI*^) [11] were also used in this study. Further, to address potentially incomplete *Celf1* deletion in *Celf1*^*cKO/cKO*^ mice, *Celf1* compound conditional knockout mice (referred to as *Celf1*^*cKO/lacZKI*^) were generated as follows. Germline *Celf1* heterozygous mice (*Celf1*^*lacZKI/+*^)were initially crossed with *Pax6GFPCre* transgenic mouse line [20] that express *Cre* recombinase in the embryonic day (E) 9.5 lens placode, to first generate *Celf1* heterozygous and *Pax6GFPCre* heterozygous (*Pax6GFPCre*^*+/-*^: *Celf1*^*lacZKI/+*^) animals. *Pax6GFPCre*^*+/-*^: *Celf1*^*lacZKI/+*^ animals were then crossed with *Celf1*^*flox/flox*^ mice to generate *Celf1* compound knockout mice (*Pax6GFPCre*^*+/-*^: *Celf1*^*flox/lacZKI*^*;* referred to as *Celf1*^*cKO/lacZKI*^) that remove *Celf1* exon five in one of the *Celf1* alleles in the developing lens starting at E9.5, while the other *Celf1* allele is germline deleted. *Celf1* conditional knockout animals (*Pax6GFPCre*^*+/-*^: *Celf1*^*flox/flox*^; referred to as *Celf1*^*cKO/cKO*^) were generated by crossing *Celf1*^*flox/flox*^ mice with *Pax6GFPCre*^*+/-*^: *Celf1*^*flox/+*^ mice. To generate *Celf1* germline knockout, *Celf1*^*lacZKI/+*^ males were crossed with either *Celf1*^*lacZKI/lacZKI*^ or *Celf1*^*lacZKI/+*^ females. Embryos were staged by designating the day that the vaginal plug was observed as embryonic day (E) 0.5. Post- natal mice were staged by designating the day of birth as post-natal day 0 (P0). Control mice used in this study were either *Celf1*^*flox/flox*^ and/or *Pax6GFPCre*^*+/-*^: *Celf1*^*+/+*^ genotype, which did not exhibit any lens defects. Genotyping was performed on tail-DNA prepared from post-natal or embryonic tissue using the Direct PCR Lysis reagent (Viagen Biotech, Los Angeles, CA). Animals were genotyped by using the following primers. *Celf1* germline deletion allele (*Celf1*^*lacZKI*^) is amplified by primers: KODEL2S-Forward-5’- GAATTATGGCCCACACCAGT-3’ and KODEL2R-Reverse-5’-GAGGGTTTTGGCTCCTATCC-3’. Celf1 floxed allele (*Celf1*^*flox*^) are amplified by primers: LF-Forward-5’- CCATAACATGAAGGTCCTCCCTGGGGT-3’ and LR-Reverse-5’- GGCTGAACTGCAGGATCACAGCACC-3’. *Cre* genomic region is amplified by primers: Forward-5’-TTCAATTTACTGACCGTACACC-3’ and 5’-CCGACGATGAAGCATGTTTAG -3’.

### Zebrafish maintenance

Wild-type AB and TL Zebrafish (*Danio rerio*) strains were used for this study. Fish were maintained at 28.5°C on a 14 hour light/10 hour dark cycle in accordance with University of Texas at Austin, IACUC provisions.

### Knockdown of *celf1* in zebrafish by splice altering morpholinos

To generate *celf1*- knockdown (KD) zebrafish (*celf1*^*KD*^), *celf1* pre-mRNA was targeted by *celf1* antisense (*celf1*-MO) and control *celf1* mismatch (*celf1*-MM) morpholinos (MOs) purchased from Open Biosystems and Gene Tools (Philomath, OR), respectively. The predicted outcome of the morpholino-mediated knockdown on the celf1 protein is a frame shift which is expected to result in 12 incorrect amino acids being translated before a premature stop codon. Both MOs were injected at a concentration of 2.2 ng/embryo at the 1–4 cell stage into wild type embryos. MOs sequences are the following: celf1-MO 5'-AACATTTTCTCACCCCTGGAAGAAT-3' (Celf1- specific morpholino (MO), test) and celf1-MM 5'-AAGATTTTGTCACCGCTGCAACAAT-3' (Celf1 mismatch morpholino (MM), control), wherein underline depicts nucleotide mismatches in the MM compared to the control. To confirm the splice-altering efficacy of the morpholino, RT-PCR was performed on both groups of injected zebrafish embryos (*celf1*-MO and *celf1*-MM) using the *celf1-*specific primers, Forward 5'- ATGAATGGGTCTCTGGACCAC-3' and Reverse 5'-CATTGTTTTTCTCACTGTCTGCAGG-3'. To confirm splice-defect induced *celf1* knockdown, DNA from the appropriate size bands isolated by agarose gel electrophoresis was purified using QIAquick Gel Extraction Kit (Qiagen, Hilden, Germany) and validated by the Sanger sequencing.

### Zebrafish *celf1* transgenics

A ~1.2kb (1152 bp) *celf1* genomic (potential enhancer) sequence that is located in the upstream region of *celf1* start codon was PCR amplified using the following primers; Forward: 5ʹ-GTACAGGTACCGCTTTCTCTTCCTGC-3ʹ and Reverse: 5ʹ-GTAGACACTAGTTTCTTCAGGCCTTC-3 and the amplicon was cloned into the Pgem-T Easy Vector (Promega, Madison, WI). This genomic (potential enhancer) region encompasses the celf1 5’ UTR and extends from +9808 to +10959 downstream of the transcription initiation site (+1) (Ensemble genome sequence (ID: ENSDARG00000005315)). This region begins at position 85 bp downstream from the start of exon 3 and includes 39bp of exon 3 as well as the first 1113 bp of intron 3. The start codon (ATG) of the zebrafish *celf1* is located in exon 4. A GFP expression vector was then constructed using the *celf1* 1.2kb genomic sequence, nuclear EGFP and SV40 polyA sequence in a Tol2 transposon as previously described [55] and according to the manufacturer’s instruction from the MultiSite Gateway Three Fragment Vector Construction Kit (Invitrogen, Carlsbad, CA). For transgenesis, 25pg (picograms) of DNA expression construct and 25pg of transposase mRNA were injected into one-cell stage embryos using a microinjector (Harvard Apparatus, Medical Systems Research Products, Holliston, MA). Injected embryos were examined under a fluorescence microscope (Leica Microscope MZ 16F, Buffalo Grove, IL) at various developmental stages to assess for expression of the EGFP reporter gene. EGFP injected embryos (F0; founder fish) were grown up 3–4 months. F0 fish harboring the transgene were mated with wild type fish to generate transgenic stable lines (F1).

### Frog *celf1*-knockdown

*Xenopus laevis* embryo manipulations, embryo staining and histological methods were performed as previously described according to approved protocols [21]. Briefly, the *celf1*-specific morpholinos 5’-TTGTGCCATTCATTATCTTAGAAAT- 3’ and 5’-ATTGTGCCATTCATTTTCTTGGAAA-3’ (NCBI Accession number BG513033), were used for generating *celf1*-knockdown. The specific nucleotides complementary to the ATG initiation codon are underlined. The morpholino 5’-CCTCTTACCTAGTTACAATTTATA- 3’ was used as standard control.

### Nuclear staining of zebrafish *celf1*-MO and c*elf1*-MM injected embryonic eye tissue

To assay the nuclear degradation defects in *celf1* knockdown embryos, 4-5dpf embryos were fixed and sectioned as previously described [56]. Sections were rehydrated with PBTD (0.1% Tween-20, 1% DMSO in 1X PBS) and nuclei staining solution (SytoxGreen, Molecular probe, Eugene, Oregon) was added at 1:1000 dilution and incubated overnight at 4°C. Slides were washed three times with PBTD and mounted with vectashield mounting media (Vector Laboratory Inc, Burlingame, CA) and imaged with confocal microscopy.

### *In situ* hybridization

*In situ* hybridization (ISH) for detecting RNA was performed as previously described for mouse [17] using *Celf1* open reading frame (ORF)-specific probe (amplified by the primers: Celf1-F-5’- GCTATTTAGGTGACACTATAGACCCTGAGCAGCCTCCACCC-3’, Celf1-R-5’- TTGTAATACGACTCACTATAGGGGCCACTGCTGCCCAGACCAC-3’, where the underlined nucleotides in the forward primer denote the SP6 promoter sequence while the underlined nucleotides in the reverse primer denote the T7 promoter sequence). Mouse E12.5 embryonic head tissue fixed overnight in 4% paraformaldehyde (PFA) at 4°C were used for obtaining coronal sections (16 μm thickness, cryosectioned) that were used for ISH analysis. For zebrafish, a *celf1* full-length ORF-specific probe was used according to an established *in situ* protocol [57]. Zebrafish eyes from 1–4 dpf embryos were fixed and cryosectioned for ISH analysis.

### Immunofluorescence

Mouse head tissue from developmental stages E11.5, E14.5, E16.5 and P0 was fixed in 4% PFA for 30 minutes on ice, and equilibrated in 30% sucrose overnight at 4°C before mounting in OCT (Tissue-Tech, Doral, FL). Sections (16um thickness) at similar depths were used in all the experiments to compare the expression of proteins between *Celf1*^*cKO/lacZKI*^ and control lens. Frozen sections (16 μm thickness) were blocked in either 5% chicken serum (Abcam, Cambridge, UK) or 5% donkey serum (Jackson ImmunoResearch, West Grove, PA) or 10% BSA (Sigma-Aldrich, St.Louis, MO) along with 0.1% Tween for one hour at room temperature. The following primary antibodies were purchased from Abcam, Cambridge, UK and Santa Cruz Biotechnology, Dallas, TX and used in the given dilutions in the respective blocking buffers: Celf1 (ab-9549, 1:500 dilution), p27^Kip1^ (SC- 528, 1:100), Lamin A/C (ab-58528, 1:100 dilution), p21^Cip1^ (SC-397, 1:100). In addition, a previously generated polyclonal antibody raised against *X*. *laevis* celf1 [21], was used at 1:500 dilution. After one hour blocking, the sections were incubated with the primary antibody overnight at 4°C. Slides were washed and incubated with the appropriate secondary antibody conjugated to Alexa Fluor 594 (1:200) (Life Technologies, Carlsbad, CA) and the nuclear stain DRAQ5 (1:2000) (Biostatus Limited, Loughborough, UK). Slides were washed, mounted using mounting media as described [58]. For F-actin staining, mouse lens tissue at P0 from both control and *Celf1*^*cKO/lacZKI*^ animals were blocked with 2% BSA (Sigma-Aldrich, St.Louis, MO) for one hour at room temperature and stained with Alexa Fluor 568 labeled phalloidin at 1:200 dilution (A12380, Invitrogen, Carlsbad, CA), 0.25% Triton X-100, and DAPI at 1:2000 dilution (D21490, Invitrogen, Carlsbad, CA) for overnight at 4°C. Lenses were washed three times with 1X PBS containing 0.1% Triton X-100 and mounted using mounting media as described. All sections were imaged using the Zeiss LSM 780 confocal microscope configured with Argon/Krypton laser (488 nm and 561 nm excitation lines) and Helium Neon laser (633 nm excitation line) (Carl Zeiss Inc, Oberkochen, Germany). Optimal adjustment of brightness/contract was performed in Adobe Photoshop (Adobe, San Jose, CA) and applied consistently for all images. Fiji imageJ software (NIH, Bethesda, MD) was used to quantify the differences in the fluorescence signal intensity of Lamin A/C and p27^Kip1^ between control and *Celf1*^*cKO/lacZKI*^ lens. To measure the fluorescence intensity, images were split into single channel and the fluorescence intensity of the region of interest (individual nuclei) was measured in the red channel or the blue channel (Draq5 staining) for normalization and quantification of the intensity ratios in three biological replicates and a student *t*-test was performed to estimate statistical significance.

### Western blot analysis

Lenses from control and *Celf1*^*cKO/lacZKI*^ animals were dissected and homogenized in ice-cold lysis buffer (50mM Tris-HCl at pH 8, 150mM NaCl, 1% nonidet P40, 0.1%SDS, 0.5% sodium deoxycholate, along with protease inhibitors (Thermo Fisher Scientific, Waltham, MA). For cell line lysate preparation, lysis buffer (1 mL) was directly added to the cell culture plate and incubated at 4°C for 30 min. Cell debris was removed by centrifuging lysates at 14,000 RPM for 30 min at 4°C. Protein concentration was determined using Pierce BCA protein kit (Thermo Fisher Scientific, Waltham, MA) according to the manufacturer’s instructions. Total protein (25–50 μg) was resolved on TGX stain free polyacrylamide gels (Bio-Rad, Hercules, CA Hercules, CA) and transferred onto PVDF membrane (Thermo Fisher Scientific, Waltham, MA). Blots were blocked with 5% non-fat dry milk for 1 hour at room temperature and incubated with primary antibody (p27^Kip1^ BD Bioscience, San Jose, CA) 610241 and Celf1 ab-9547 at 1:500 and 1:1000 dilutions, respectively) over night at 4°C. Blots were incubated with secondary antibodies conjugated to horseradish peroxidase (Cell Signaling Technology, Danvers, MA) for one hour at room temperature, and the signals were detected with SuperSignal^TM^ West Femto Maximum Sensitivity Substrate (Thermo Fisher Scientific, Waltham, MA).

### Dark-field microscopy, grid imaging and histological analysis

Control and *Celf1* deficient mouse eyes were dissected in 1X PBS and imaged by light microscopy (Zeiss Stemi SV dissecting microscope). Mouse eyes were further dissected to isolate lenses for grid imaging. For the grid images, lenses from control and *Celf1* deficient mice at stage P30 were placed on a 300-mesh electron microscopy grid (Electron Microscopy Sciences, Hatsfield, PA, Catalog No. 6300H-Cu) and imaged to evaluate the refractive properties of the lens as previously described [18]. Hematoxylin and Eosin (H&E) staining was performed on sections from mouse embryonic head tissue or postnatal eye tissue as described [58].

### Scanning electron microscopy

Mouse eyes from control and Celf1 deficient mice at stage P15 were processed as previously described [18]. Samples were imaged with a field emission scanning electron microscope, Hitachi S-4700 (Tokyo, Japan).

### RNA isolation and RT-qPCR

Four lenses for each of three biological replicates were collected from control and *Celf1* deficient mice at stage P0 and total RNA was extracted using RNeasy mini kit (Qiagen, Hilden, Germany). cDNA synthesis and RT-qPCR was performed as described [18] on ABI7300 Real-Time PCR system (Applied Biosystems, Foster City, CA) using Power Syber Green PCR master mix (Invitrogen Life technologies, Carlsbad, CA). Transcript levels were normalized to the housekeeping gene *Gapdh*. For each sample, differential expression was determined using ΔΔCT method. The following primers were used for qRT-PCR: Celf1-F-5’-ACAGATGAAGCCTGCTGACA-3’ and Celf1-R-5’-CTCTGCTCAAGCCATCAGGT-3’; Dnase2b-F-5’-TGCTCTGGGGAGGACCTTAC-3’ and Dnase2b-R-5’-CCCCTGCGTTCTGTTCCATA-3’; p27^Kip1^-F-5’-GCCAGACGTAAACAGCTCCGAATT-3’ and p27^Kip1^-R-5’-AGGCAGATGGTTTAAGAGTGCCT-3’; p21^Cip1^-F-5’-CGGTGTCAGAGTCTAGGGGA-3’ and p21^Cip1^-R-5’-CATGAGCGCATCGCAATCAC-3’; Actn2-F-5’-GAATCAGATAGAGCCCGGCG-3’ and Actn2-R-5’-ATGTTCTCGATCTGGGTGCC-3’; Sptb-001 ENSMUST00000021458—Exon 36 -F-5’-TGGCTACAGAGCATGAGCAC-3’ and Exon 36 -R-5’-TCCTTTTCCTTCTTGCCAAC-3’; Sptb-002 ENSMUST 00000166101- Exon 32-F-5’- AGAGGAGGAAGGCGAGACAG-3’ and Exon 32-R-5’-GGAACTAGACAAGCGGGACA-3’; Gapdh-F-5’-GATCGTGGAAGGGCTAATGA-3’ and Gapdh-R-5’-GACCACCTGGTCCTCTGTGT-3’; B2M-F-5’- TGGTGCTTGTCTCACTGACC-3’and B2M-R-5’- CCGTTCTTCAGCATTTGGAT-3’. Primers for Sptb isoforms RT-PCR: Sptb-001 ENSMUST00000021458 Exon 32-36-F-5’-AGAGGAGGAAGGCGAGACAG-3’ and Sptb-001 ENSMUST00000021458 Exon 32-36-R-5’-TCCTTTTCCTTCTTGCCAAC-3’; Sptb-002 ENSMUST00000166101 Exon 32-F-5’-AGAGGAGGAAGGCGAGACAG-3’ and Sptb-002 ENSMUST00000166101 Exon 32-R-5’-CTCTGGCAGCAGCGACTC-3’. For fish, total RNA from 1dpf embryos was isolated using Trizol reagent (Invitrogen Life technologies, Carlsbad, CA), and cDNA was synthesized using iScript cDNA synthesis kit (Bio-Rad, Hercules, CA).

### Microarray analysis

Total RNA from wild-type and *Celf1*_*cKO/lacZKI*_ mouse lenses at stage P0 was isolated (two lenses for each biological replicate) as described above and used for microarray gene expression profiling analysis on the Illumina MouseWG-6 v2.0 BeadChip platform (Illumina, San Diego, CA). Raw microarray data files were imported to ‘R’ statistical environment (http://www.r-project.org/) and background corrected using lumi package at Bioconductor (www.bioconductor.org), followed by normalization by Rank Invariant method as previously described [59]. Differentially expressed genes (DEGs) were identified at a significant *p*-value < 0.05 and fold change cut-off of ±2.0 or ±2.5. High-priority candidates were identified using *iSyTE*-based analysis and previously established filtering criteria [22,59]. The microarray data generated in this study are deposited in the Gene Expression Ominbus database (www.ncbi.nih.gov/geo) and the accession number is GSE101393.

### *iSyTE*-based analysis

*Celf1* transcript expression in developing lens was investigated in previously generated wild-type mouse lens microarrays (E10.5, E11.5, E12.5) (GSE32334) in the *iSyTE* database [17]. Scatter plot analysis was performed, as previously described [59], to investigate if DEGs in *Celf1*^*lacZKI/lacZKI*^ lens microarrays showed preferential expression in the lens. Briefly, *iSyTE* assigns lens-enrichment scores to genes based on their comparison with mouse whole embryonic tissue (WB) [17]. For *iSyTE* lens- enrichment (fold-change), P0 lens microarrays (GSE165333) were compared to WB (GSE32334). In a quadrant plot, *Celf1*^*lacZKI/lacZKI*^ lens DEGs (±2.0 fold-change, *p*-value < 0.05) were plotted against their *iSyTE* lens enrichment fold-change.

### RNA immunoprecipitation

Celf1/RNA-immunoprecipitation was performed according to manufacturer instructions (EMD Millipore, Billerica, MA, 17–700). Briefly, wild-type P15 mouse lens lysates were used (*n* = 15 P15 stage lenses per replicate). Pre-conjugation of Celf1 antibody (EMD Millipore, Billerica, MA, 03–104) and IgG antibody with magnetic beads was performed for 45 min. at room temperature and unbound antibody was removed by washing. Lens protein lysate was added to the beads-antibody complex and incubated overnight at 4°C. Bound RNA isolated by phenol-chloroform extraction was used in RT-PCR analysis.

### Cross-Linking immunoprecipitation

Freshly dissected wild-type stage P13 mouse lenses were UV irradiated three times at 4000 μJ/cm^2^ and 254 nm on ice for cross-linking, and stored at −80°C. Celf1/RNA complexes were immunoprecipitated from lens protein extracts as described [60], except that the RNase treatment was omitted. The co- immunoprecipitated RNA was analyzed by RT-qPCR. B2M (Beta-2-Microglobulin) is used as a negative control in CLIP-RTqPCR and RIP-RTqPCR experiments because B2M mRNA levels are not affected by Celf1 inactivation and there is no evidence of an interaction between B2M and Celf1.

### Cell culture

The mouse lens epithelial cell line 21EM15 was directly obtained from Dr. John Reddan (Oakland University, MI). Cells were cultured in 100mm cell culture treated plates (Eppendorf) under standard conditions (10 mL of DMEM with 4.5 g/L glucose, L- glutamine, and sodium pyruvate included (Corning Cellgro, Manassas, VA, 10-013-CV)), 10% Fetal Bovine Serum (Fisher Scientific, Pittsburg, PA, 03-600-511), and 1% penicillin- streptomycin (GE Healthcare Life Sciences, Logan, UT, SV30010). Cells were incubated at 37°C in a humid chamber with 5% CO2 as described [16].

### Generation of *Celf1* stable knockdown cell lines

Five different lentiviral particles containing shRNA (small hairpin RNA) sequences targeting mouse Celf1 mRNA (Sigma-Aldrich, St-Louis, MO, clone ID: NM_198683.1-1279s21c1; NM_198683.1-869s21c1; NM_198683.1- 1739s21c1; NM_198683.1-1320s21c1; NM_198683.1-868s21c1) were used to transduce mouse lens cell line 21EM15 as previously described [16]. Non-targeting shRNA control transduction particles were used as a control (Sigma-Aldrich, Catalog No. SHC0-016V- 1EA). Standard manufacturer protocol was used to infect 21EM15 cells with lentiviral particles. Briefly, anti-Celf1 shRNA containing viral particles (10^6^TU) were used to infect 1.6 x 10^4^ 21EM15 cells in the presence of 8 μg/ml polybrene (Sigma, St-Louis, MO). Media was changed 24 hours after transduction to prevent cell death from virus toxicity. Clones were selected at a final concentration of 6μg/ml puromycin and clones were selected using Pyrex cloning cylinders (Sigma-Aldrich). The extent of Celf1 knockdown was determined by Western blot analysis.

### Luciferase reporter assays

To test the Celf1 mediated translational repression of p27^Kip1^, a plasmid containing p27^Kip1^ 5’UTR sequence upstream of firefly luciferase reporter, pGL3- p27^Kip1^ LUC (Addgene original plasmid # 23047) and an internal control vector, pRL-TK *Renilla* luciferase (Promega) were transiently co-transfected into *Celf1* knockdown and control 21EM15 lens cell lines. After 48 hours of transfection cells were collected and firefly luciferase and *Renilla* luciferase activity was measured using Promega Dual luciferase reporter assay system (Promega, Madison, WI, E1910) according to the manufacturer’s instructions. Signals were measured with the PromegaTM GloMaxTM 20/20 Luminometry System (Promega, Madision, WI).

### Celf1 over-expression assays

To study Celf1 mediated regulation of Dnase2b mRNA, reporter constructs of (a) Dnase2b 3’UTR and (b) Celf1 ORF (Celf1 over-expression) were generated. To generate the Dnase2b 3’UTR plasmid, wild-type Dnase2b 3’UTR was cloned downstream of the firefly luciferase gene in the pmirGlo vector (Promega, Madision, WI) using the Gibson Assembly Master Mix kit (New England Biolabs, Ipswich, MA, NEB#E2611S/L) with the following primers: Forward-5’-tagttgtttaaacgagctCACACCCTCTGTCCTTGAA-3’ and Reverse-5’-atgcctgcaggtcgactCCTATATTTATTCACTTCCTTTACTGTC-3’. The nucleotides corresponding to the target vector for the Gibson assembly are in lowercase. To generate the Celf1 over-expression plasmid, the Gateway cloning system (Thermo Fisher Scientific, Waltham, MA) was used. Briefly, the full-length coding sequence of Celf1 flanked by *attb* sites was generated by PCR according to the manufacturer’s instructions using the following primers: Forward- 5’-GGGGACAAGTTTGTACAAAAAAGCAGGCTTCACCAT-3’ and Reverse-5’- GGGGACCACTTTGTACAAGAAAGCTGGGTCCTATCAGTAGGGCTTACTATCATTCTTGGATGGC TGCGTTTAAGTTGGATT-3’. The PCR product was used in BP recombination reaction with *attp* containing pDONR221 vector (Thermo Fisher Scientific, Waltham, MA) to create the entry vector. Next, using LR recombination reaction, the *Celf1* gene from BP clone was moved into a destination vector, pDEST47 (Thermo Fisher Scientific, Waltham, MA) and confirmed by Sanger Sequencing. Celf1 over-expression plasmid was transfected into NIH3T3 cells for up to 72 hours and cells were assayed for Celf1 elevated levels by Western blotting. For over- expression assays, both Celf1 over-expression plasmid and the dual luciferase-Dnase2b 3’UTR plasmid were transiently transfected into NIH3T3 cells for 48 hours. Cells were collected, total RNA was isolated and cDNA were synthesized, and RT-qPCRs were performed as described above using the following primers to amplify the luciferase product: Firefly Luc Forward-5’-GCCCCAGCTAACGACATCTA-3’, Firefly Luc Reverse-5’- TCTTTTGCAGCCCTTTCTTG-3’.

### Statistical analysis

All experiments were performed in three biological replicates unless stated otherwise. Statistical significance for RT-qPCR data was determined using nested ANOVA as previously described [18]. Statistical significance for the fluorescence intensity measurement and luciferase assays was determined by two-tailed student t-test.

## Supporting information

S1 Fig *Celf1* mRNA expression in vertebrate lens development. **(A)** *iSyTE* identifies *Celf1* as a highly lens-enriched gene in lens development. Lens enrichment extent is indicated by differing red color intensities in the heat-map. **(B)** Analysis of *iSyTE* lens microarray datasets from mouse embryonic stages 10.5, 11.5 and 12.5 shows highly enriched expression of *Celf1* compared to whole embryonic body tissue (WB) control. *Celf1* microarray probe binding fluorescent signal intensity is represented on the Y-axis and the different mouse embryonic lens stages are shown on the X-axis. **(C)** At 2dpf (day post fertilization), **(D)** 3dpf and **(E)** 4dpf, zebrafish lenses exhibit expression of *celf1* mRNA in the transition zone (arrow in C) and in later stages in the posterior region (arrows in D and E). (**C’** to **E’**) High-magnification of C to E. **(F)** In *Xenopus laevis*, *celf1* mRNA expression is observed from early developmental stage (St. 23) in the eye region (arrow; lens area indicated by broken white line). **(F’)** High-magnification of F. Lens area is indicated by broken white line. Asterisks in B represents a *p*-value less than 0.05.
(TIFF)

S2 Fig *celf1* reporter gene analysis and protein expression in developing vertebrate lens. **(A)** Schematic of the zebrafish *celf1* gene (not drawn to scale) shows the location of the ~1.2kb potential enhancer in the genomic region upstream of the start codon (which is located in exon 4). This ~1.2kb *celf1* genomic region is fused to EGFP in the plasmid construct that is used in the reporter assays. **(B** to **E’)** Lens-specific expression of EGFP in zebrafish indicates strong *celf1* enhancer activity at (B, B’) 1dpf, (C) 2dpf, (D) 3dpf and (E, E’) 4dpf. **(F** to **I”)** Transverse sections of zebrafish eye exhibit high EGFP expression at (F to F”) 1dpf, (G to G”) 2dpf, (H to H”) 3dpf and (I to I”) 4dpf. **(J)** In *Xenopus laevis*, at St. 27, high celf1 protein expression is present. **(J’)** High-magnification of dotted line area in J. Dotted-line circle shows lens region. **(K** and **L)** In mouse lens, high Celf1 protein expression is detected by a mouse monoclonal Celf1 antibody at stages E11.5 and E14.5. Fiber cells (f) and epithelium (e). **(K’** and **L’)** High-magnification of dotted-line area in K and L, respectively. **(M-P”‘)** In mouse lens, rabbit Celf1 antibody detects Celf1 protein in lens development at stages E12.5, E14.5, E16.5 and P0. High magnification in dotted area is shown. In early stages, Celf1 protein is detected predominantly in fiber cells (f), and at later stages, while it retains high fiber cell expression, it is also detected in the epithelium (e). **(Q)** *Celf1*^*lacZKI/+*^ mouse reporter analysis reveals β-galactosidase activity in the lens at embryonic stage E11.5, indicative of endogenous *Celf1* promoter/enhancer driven gene expression. **(Q’)** High-magnification of eye region in M’ shows high β-galactosidase activity in the lens (arrow). Scale bar in K and L is 75 μm while in K’ and L’ is 12 μm.
(TIFF)

S3 Fig Generation of the *Celf1* conditional knockout mice. **(A)** Schematic representation of targeting strategy to generate *Celf1* conditional knockout (*Celf1*^*cKO/cKO*^) and *Celf1* compound conditional (*Celf1*^*cKO/lacZKI*^) mice. The “conditional allele” represents *Celf1* floxed allele wherein, exon five is flanked by *loxP* (red arrowheads). The “after *Cre* recombination allele” shows the rearranged *Celf1* allele after Cre mediated exon five deletion. The “*Celf1* germline KO allele” (*Celf1*^*lacZKI*^) represents the *Celf1* germline targeted allele that has the *lacZ* cassette inserted in exon one as previously described [11]. Black arrows indicate position of genotyping primers. **(B)** *Pax6GFPCr*e transgenic mouse line carries a *GFP-Cre* fusion gene driven by the *Pax6 P0* promoter 3.9-kb upstream region and show GFP-Cre expression early in lens development starting in the lens placode stage at embryonic stage E9.5. Strong GFP-Cre is observed in the lens vesicle at E10.5. **(C)** PCR analysis confirms the deletion of the floxed exon five in lens DNA obtained from *Celf1*^*cKO/lacZKI*^ mice. The *Celf1 lacZ* knock-in allele is as previously described [11]. **(D)** RT-qPCR analysis confirms significantly reduced (~25-fold) *Celf1* mRNA levels in P0 *Celf1*^*cKO/lacZKI*^ lens. **(E)** Compared to control, immunofluorescence analysis with and without Draq5 staining of DNA shows the near absence of Celf1 protein in *Celf1*^*cKO/lacZKI*^ lens at E14.5. **(F)** Western blot analysis shows the absence of Celf1 protein in *Celf1*^*cKO/lacZKI*^ lenses at P30, confirming *Celf1* deletion in the mouse lens. Asterisks in D represent a *p*-value of less than 0.005. Scale bar in E is 12 μm.
(TIFF)

S4 Fig Knockdown of *celf1* in zebrafish and *Xenopus laevis*. **(A)** Schematic representation of normal and altered splicing of *celf1* in zebrafish. *celf1* morphants (*celf1* MO) were generated by using splice altering morpholinos to knockdown *celf1* in zebrafish. **(B)** Schematic representation of PCR strategy to detect normal (310 bp) and altered splicing (227 bp) in zebrafish. **(C)** RT-PCR showing a 310 bp band indicating normal splicing in *celf1* controls (*celf1 MM*) and a 227 bp band in the *celf1*-knockdown (*celf1* MO) embryos confirming splice altering activity in zebrafish morphants. **(D)** Sequencing data confirms the exclusion of exon five in zebrafish *celf1* MO embryos but not in controls. **(E)** In *X*. *laevis*, *celf1* KD animals were generated by injecting morpholinos against *celf1* (see methods) in one of the cells of the embryos at the two-cell-stage, as described previously [21].
(TIFF)

S5 Fig *celf1* deficiency results in ocular defects in zebrafish and *Xenopus laevis*. **(A)** *celf1* knockdown (KD) in zebrafish results in microphthalmia and clouding of the eye by 4dpf. **(B** and **C)** Histological analysis of *X*. *laevis* morphants at St. 42. *celf1*-knockdown (morpholino injected side) shows various eye defects such as (B) small eye and (C) skewed eye, that are absent in the control uninjected side. **(D)** Graph representing the distribution of the observed eye defects in *X*. *laevis celf1* morphants from three independent experiments.
(TIFF)

S6 Fig *Celf1* deletion in mouse causes severe lens defects and cataract. **(A** to **B’)** Six weeks (A, A’) and four month (B, B’) aged *Celf1*^*lacZKI/lacZKI*^ lenses shows severe cataracts and disruption of the lens tissue compared to control. In A’, the dotted area represents the disintegrated lens tissue around the remaining lens core in *Celf1*^*lacZKI/lacZKI*^ mice. **(C** to **C’)** Histological analysis of *Celf1*^*cKO/cKO*^ lens exhibits slightly delayed fiber cell elongation compared to the control lens at stage E12.5. Scale bars in C, C’ are 100 μm.
(TIFF)

S7 Fig Xenopus *celf1* morphants exhibit segmentation defects. Previously, Xenopus *celf1* morphants have been described to have defective somite segmentation. In our present study on Xenopus, we also observe these defects. The morpholino injected side (left) of Xenopus embryos (St. 42) shows somite segmentation defects (arrow).
(TIFF)

S8 Fig *Celf1*^*cKO/cKO*^ mice exhibit nuclear degradation defects. **(A)** Z-stack serial imaging analysis (serial slices#5 through 8 in a series of 10) demonstrates that *Celf1*^*cKO/cKO*^ mouse lens at stage P0 exhibits presence of nuclei (asterisk) in centrally located fiber cells compared to control mouse lens which shows a distinct nuclei-free zone in this fiber cell region (broken white line). **(B)** Z-stack serial imaging analysis (serial slices#7 through 12 in a series of 22) demonstrates that *Celf1*^*cKO/cKO*^ mouse lens at stage P0 exhibits presence of nuclei (asterisk) in centrally located fiber cells compared to control mouse lens which shows a distinct nuclei-free zone in this fiber cell region (broken white line).
(TIFF)

S9 Fig *Celf1* deficiency-mediated nuclear degradation defects are persistent. **(A** to **B”)** In mice, compared to control, *Celf1*^*cKO/cKO*^ lens at stage P0 exhibits fiber cell nuclear degradation defects. Note the abnormal presence of nuclei (asterisks) in the central region of the fiber cells. A’ to B” are higher magnification images of A and B, respectively. **(C** to **D”)** Compared to control, *Celf1*^*lacZKI/lacZKI*^ lens at seven-week age continue to exhibit nuclear degradation defects. Note the abnormal presence of nuclei (asterisks) in the central fiber cell region. C’ to D” are higher magnification images of C and D, respectively. (**E** and **F**) Compared to control where a clear nuclear free zone is visible (broken white line), *Celf1*^*lacZKI/lacZKI*^ mouse lens at 4 months continue to exhibit nuclear degradation defects. Note the abnormal presence of nuclei (asterisks) in the central fiber cell region. **(G** to **H’)** Compared to control, zebrafish *celf1* KD lens exhibits nuclear degradation defects at stage 5dpf. Note the abnormal presence of nuclei (asterisks) in the central fiber cell region. E’ and F’ are high-magnification of the dotted-line area in E and F.
(TIFF)

S10 Fig Celf1 over-expression in NIH3T3 cells. **(A)** Dual reporter construct of Dnase2b 3’UTR-firefly luciferase and *Renilla* luciferase. Celf1 CDS-GFP fusion expression construct. **(B)** Western blot analysis confirms over-expression of Celf1 protein at 24 hr, 36 hr, and 48 hr after the transfections compared to the control (48 hr) that was transfected with an empty vector.
(TIFF)

S11 Fig *Celf1*^*cKO/lacZKI*^ lens exhibits reduction in phosphorylated-lamin A/C in fiber cells. (**A, A’**) In *Celf1*^*cKO/lacZKI*^ lens at stage P0, phosphorylation of Lamin A/C (pLamin A/C) is reduced compared to control. High-magnification of fiber cell regions (dotted-line areas) of control (**B**-**E**) and *Celf1*^*cKO/lacZKI*^ (**B’**-**E’**) lens are shown. Arrows in B-D indicate high phosphorylated lamin A/C expression while its reduced expression in B’-E’ is indicated by asterisks. (**F)** In mouse stage P0 lens, quantification of the immunofluorescence signals shows significantly reduced Lamin A/C protein levels in *Celf1*^*cKO/LacZKI*^ lens compared to control. Asterisk in F represents a *p*-value <0.05. Scale bar represents 75 μm.
(TIFF)

S12 Fig Celf1 negatively regulates p27^Kip1^ in lens development. **(A)** At E14.5, the p27^Kip1^ protein levels are reduced in central fiber region in the control lens–as expected, while in the *Celf1*^*cKO/lacZKI*^ lens, p27^Kip1^ protein levels in the central fiber region are elevated. **(B)** At E16.5, *Celf1*^*cKO/lacZKI*^ lens epithelium exhibits elevated p27^Kip1^ protein in a few nuclei compared to control, but this difference was not observed at stage P0. **(C, D)** At stages E16.5 and P0, both control and *Celf1*^*cKO/lacZKI*^ lenses show p27^Kip1^ protein expression in the transition zone and in cortical fiber cells. However, p27^Kip1^ protein expression is distinctly elevated in the centrally located fiber cells in *Celf1*^*cKO/lacZKI*^ lenses compared to control. Broken white line areas of fiber cell regions is shown at high-magnification. Scale bar represents 75 μm.
(TIFF)

S13 Fig Celf1-knockdown in mouse lens epithelial cell line 21EM15. Western blot analysis confirms the reduction of Celf1 protein in the lenti-viral-based stable *Celf1* knockdown lens cell line 21EM15, compared to control.
(TIFF)

S14 Fig *Celf1*^*cKO/LacZKI*^ lenses exhibit differential expression of Sptb isoforms. RT-PCR analysis indicates that the high-abundant Beta-spectrin (*Sptb*) isoform (isoform 1 (ENSMUST00000021458)) is reduced, while the low-abundant isoform (isoform 2 (ENSMUST00000166101)) is abnormally elevated in *Celf1*^*cKO/lacZKI*^ lenses. Amplicon sizes of *Sptb* isoform 1 and isoform 2 are 681 and 121 base-pairs, respectively.
(TIFF)

## References

[1] Singh G, Pratt G, Yeo GW, Moore MJ. The Clothes Make the mRNA: Past and Present Trends in mRNP Fashion. Annu Rev Biochem. 2015;84: 325–354. doi: 10.1146/annurev-biochem-080111-092106 25784054 10.1146/annurev-biochem-080111-092106 PMC4804868

[2] Cvekl A, Ashery-Padan R. The cellular and molecular mechanisms of vertebrate lens development. Development. 2014;141: 4432–4447. doi: 10.1242/dev.107953 25406393 10.1242/dev.107953 PMC4302924

[3] Dash S, Siddam AD, Barnum CE, Janga SC, Lachke SA. RNA-binding proteins in eye development and disease: implication of conserved RNA granule components. Wiley Interdiscip Rev RNA. 2016;7: 527–557. doi: 10.1002/wrna.1355 27133484 10.1002/wrna.1355 PMC4909581

[4] Gerstberger S, Hafner M, Tuschl T. A census of human RNA-binding proteins. Nat Rev Genet. 2014;15: 829–845. doi: 10.1038/nrg3813 25365966 10.1038/nrg3813 PMC11148870

[5] Cvekl A, Zhang X. Signaling and Gene Regulatory Networks in Mammalian Lens Development. Trends Genet. 2017; doi: 10.1016/j.tig.2017.08.001 28867048 10.1016/j.tig.2017.08.001 PMC5627649

[6] Lachke SA, Maas RL. RNA Granules and Cataract. Expert Rev Ophthalmol. 2011;6: 497–500. doi: 10.1586/eop.11.53 23847690 10.1586/eop.11.53 PMC3705770

[7] Wolf L, Gao CS, Gueta K, Xie Q, Chevallier T, Podduturi NR, et al Identification and characterization of FGF2-dependent mRNA: microRNA networks during lens fiber cell differentiation. G3 (Bethesda). 2013;3: 2239–2255. doi: 10.1534/g3.113.008698 24142921 10.1534/g3.113.008698 PMC3852386

[8] Dasgupta T, Ladd AN. The importance of CELF control: molecular and biological roles of the CUG-BP, Elav-like family of RNA-binding proteins. Wiley Interdiscip Rev RNA. 2012;3: 104–121. doi: 10.1002/wrna.107 22180311 10.1002/wrna.107 PMC3243963

[9] Vlasova-St Louis I, Dickson AM, Bohjanen PR, Wilusz CJ. CELFish ways to modulate mRNA decay. Biochim Biophys Acta. 2013;1829: 695–707. doi: 10.1016/j.bbagrm.2013.01.001 23328451 10.1016/j.bbagrm.2013.01.001 PMC3640684

[10] Barreau C, Paillard L, Méreau A, Osborne HB. Mammalian CELF/Bruno-like RNA-binding proteins: molecular characteristics and biological functions. Biochimie. 2006;88: 515–525. doi: 10.1016/j.biochi.2005.10.011 16480813 10.1016/j.biochi.2005.10.011

[11] Kress C, Gautier-Courteille C, Osborne HB, Babinet C, Paillard L. Inactivation of CUG-BP1/CELF1 causes growth, viability, and spermatogenesis defects in mice. Mol Cell Biol. 2007;27: 1146–1157. doi: 10.1128/MCB.01009-06 17130239 10.1128/MCB.01009-06 PMC1800704

[12] Philips AV, Timchenko LT, Cooper TA. Disruption of splicing regulated by a CUG-binding protein in myotonic dystrophy. Science. 1998;280: 737–741. 9563950 10.1126/science.280.5364.737

[13] Giudice J, Xia Z, Wang ET, Scavuzzo MA, Ward AJ, Kalsotra A, et al Alternative splicing regulates vesicular trafficking genes in cardiomyocytes during postnatal heart development. Nat Commun. 2014;5: 3603 doi: 10.1038/ncomms4603 24752171 10.1038/ncomms4603 PMC4018662

[14] Chaffee BR, Shang F, Chang M-L, Clement TM, Eddy EM, Wagner BD, et al Nuclear removal during terminal lens fiber cell differentiation requires CDK1 activity: appropriating mitosis-related nuclear disassembly. Development. 2014;141: 3388–3398. doi: 10.1242/dev.106005 25139855 10.1242/dev.106005 PMC4199135

[15] Nishimoto S, Kawane K, Watanabe-Fukunaga R, Fukuyama H, Ohsawa Y, Uchiyama Y, et al Nuclear cataract caused by a lack of DNA degradation in the mouse eye lens. Nature. 2003;424: 1071–1074. doi: 10.1038/nature01895 12944971 10.1038/nature01895

[16] Lachke SA, Alkuraya FS, Kneeland SC, Ohn T, Aboukhalil A, Howell GR, et al Mutations in the RNA granule component TDRD7 cause cataract and glaucoma. Science. 2011;331: 1571–1576. doi: 10.1126/science.1195970 21436445 10.1126/science.1195970 PMC3279122

[17] Lachke SA, Ho JWK, Kryukov GV, O’Connell DJ, Aboukhalil A, Bulyk ML, et al iSyTE: integrated Systems Tool for Eye gene discovery. Invest Ophthalmol Vis Sci. 2012;53: 1617–1627. doi: 10.1167/iovs.11-8839 22323457 10.1167/iovs.11-8839 PMC3339920

[18] Agrawal SA, Anand D, Siddam AD, Kakrana A, Dash S, Scheiblin DA, et al Compound mouse mutants of bZIP transcription factors Mafg and Mafk reveal a regulatory network of non-crystallin genes associated with cataract. Hum Genet. 2015;134: 717–735. doi: 10.1007/s00439-015-1554-5 25896808 10.1007/s00439-015-1554-5 PMC4486474

[19] Kakrana A, Yang A, Anand D, Djordjevic D, Ramachandruni D, Singh A, et al iSyTE 2.0: a database for expression-based gene discovery in the eye. Nucleic Acids Res. 2018;46: D875–885. doi: 10.1093/nar/gkx837 29036527 10.1093/nar/gkx837 PMC5753381

[20] Rowan S, Siggers T, Lachke SA, Yue Y, Bulyk ML, Maas RL. Precise temporal control of the eye regulatory gene Pax6 via enhancer-binding site affinity. Genes Dev. 2010;24: 980–985. doi: 10.1101/gad.1890410 20413611 10.1101/gad.1890410 PMC2867212

[21] Gautier-Courteille C, Le Clainche C, Barreau C, Audic Y, Graindorge A, Maniey D, et al EDEN-BP-dependent post-transcriptional regulation of gene expression in Xenopus somitic segmentation. Development. 2004;131: 6107–6117. doi: 10.1242/dev.01528 15548579 10.1242/dev.01528

[22] Manthey AL, Terrell AM, Lachke SA, Polson SW, Duncan MK. Development of novel filtering criteria to analyze RNA-sequencing data obtained from the murine ocular lens during embryogenesis. Genomics Data. 2014;2: 369–374. doi: 10.1016/j.gdata.2014.10.015 25478318 10.1016/j.gdata.2014.10.015 PMC4248573

[23] Audette DS, Anand D, So T, Rubenstein TB, Lachke SA, Lovicu FJ, et al Prox1 and fibroblast growth factor receptors form a novel regulatory loop controlling lens fiber differentiation and gene expression. Development. 2016;143: 318–328. doi: 10.1242/dev.127860 26657765 10.1242/dev.127860 PMC4725344

[24] Cavalheiro GR, Matos-Rodrigues GE, Zhao Y, Gomes AL, Anand D, Predes D, et al N-myc regulates growth and fiber cell differentiation in lens development. Dev Biol. 2017;429: 105–117. doi: 10.1016/j.ydbio.2017.07.002 28716713 10.1016/j.ydbio.2017.07.002 PMC5586101

[25] De Maria A, Bassnett S. DNase IIbeta distribution and activity in the mouse lens. Invest Ophthalmol Vis Sci. 2007;48: 5638–5646. doi: 10.1167/iovs.07-0782 18055814 10.1167/iovs.07-0782

[26] Nakahara M, Nagasaka A, Koike M, Uchida K, Kawane K, Uchiyama Y, et al Degradation of nuclear DNA by DNase II-like acid DNase in cortical fiber cells of mouse eye lens. FEBS J. 2007;274: 3055–3064. doi: 10.1111/j.1742-4658.2007.05836.x 17509075 10.1111/j.1742-4658.2007.05836.x

[27] Zhang P, Wong C, DePinho RA, Harper JW, Elledge SJ. Cooperation between the Cdk inhibitors p27(KIP1) and p57(KIP2) in the control of tissue growth and development. Genes Dev. 1998;12: 3162–3167. 9784491 10.1101/gad.12.20.3162 PMC317217

[28] Lyu L, Whitcomb EA, Jiang S, Chang M-L, Gu Y, Duncan MK, et al Unfolded-protein response-associated stabilization of p27(Cdkn1b) interferes with lens fiber cell denucleation, leading to cataract. FASEB J. 2016;30: 1087–1095. doi: 10.1096/fj.15-278036 26590164 10.1096/fj.15-278036 PMC4750420

[29] Rowan S, Chang M-L, Reznikov N, Taylor A. Disassembly of the lens fiber cell nucleus to create a clear lens: The p27 descent. Exp Eye Res. 2017;156: 72–78. doi: 10.1016/j.exer.2016.02.011 26946072 10.1016/j.exer.2016.02.011 PMC5465823

[30] Marquis J, Paillard L, Audic Y, Cosson B, Danos O, Le Bec C, et al CUG-BP1/CELF1 requires UGU-rich sequences for high-affinity binding. Biochem J. 2006;400: 291–301. doi: 10.1042/BJ20060490 16938098 10.1042/BJ20060490 PMC1652823

[31] Vlasova IA, Tahoe NM, Fan D, Larsson O, Rattenbacher B, Sternjohn JR, et al Conserved GU-rich elements mediate mRNA decay by binding to CUG-binding protein 1. Mol Cell. 2008;29: 263–270. doi: 10.1016/j.molcel.2007.11.024 18243120 10.1016/j.molcel.2007.11.024 PMC2367162

[32] Rattenbacher B, Beisang D, Wiesner DL, Jeschke JC, von Hohenberg M, St Louis-Vlasova IA, et al Analysis of CUGBP1 targets identifies GU-repeat sequences that mediate rapid mRNA decay. Mol Cell Biol. 2010;30: 3970–3980. doi: 10.1128/MCB.00624-10 20547756 10.1128/MCB.00624-10 PMC2916446

[33] Chaudhury A, Cheema S, Fachini JM, Kongchan N, Lu G, Simon LM, et al CELF1 is a central node in post-transcriptional regulatory programmes underlying EMT. Nat Commun. 2016;7: 13362 doi: 10.1038/ncomms13362 27869122 10.1038/ncomms13362 PMC5121338

[34] Edwards JM, Long J, de Moor CH, Emsley J, Searle MS. Structural insights into the targeting of mRNA GU-rich elements by the three RRMs of CELF1. Nucleic Acids Res. 2013;41: 7153–7166. doi: 10.1093/nar/gkt470 23748565 10.1093/nar/gkt470 PMC3737555

[35] Terrell AM, Anand D, Smith SF, Dang CA, Waters SM, Pathania M, et al Molecular characterization of mouse lens epithelial cell lines and their suitability to study RNA granules and cataract associated genes. Exp Eye Res. 2015;131: 42–55. doi: 10.1016/j.exer.2014.12.011 25530357 10.1016/j.exer.2014.12.011 PMC4387128

[36] Zheng Y, Miskimins WK. CUG-binding protein represses translation of p27Kip1 mRNA through its internal ribosomal entry site. RNA Biol. 2011;8: 365–371. doi: 10.4161/rna.8.3.14804 21508681 10.4161/rna.8.3.14804 PMC3218506

[37] Parker SB, Eichele G, Zhang P, Rawls A, Sands AT, Bradley A, et al p53-independent expression of p21Cip1 in muscle and other terminally differentiating cells. Science. 1995;267: 1024–1027. 7863329 10.1126/science.7863329

[38] Baker DJ, Weaver RL, van Deursen JM. p21 both attenuates and drives senescence and aging in BubR1 progeroid mice. Cell Rep. 2013;3: 1164–1174. doi: 10.1016/j.celrep.2013.03.028 23602569 10.1016/j.celrep.2013.03.028 PMC3785294

[39] Le Tonquèze O, Gschloessl B, Legagneux V, Paillard L, Audic Y. Identification of CELF1 RNA targets by CLIP-seq in human HeLa cells. Genom Data. 2016;8: 97–103. doi: 10.1016/j.gdata.2016.04.009 27222809 10.1016/j.gdata.2016.04.009 PMC4872370

[40] Schleicher M, André E, Hartmann H, Noegel AA. Actin-binding proteins are conserved from slime molds to man. Dev Genet. 1988;9: 521–530. doi: 10.1002/dvg.1020090428 3243032 10.1002/dvg.1020090428

[41] Gupta V, Discenza M, Guyon JR, Kunkel LM, Beggs AH. α-Actinin-2 deficiency results in sarcomeric defects in zebrafish that cannot be rescued by α-actinin-3 revealing functional differences between sarcomeric isoforms. FASEB J. 2012;26: 1892–1908. doi: 10.1096/fj.11-194548 22253474 10.1096/fj.11-194548 PMC3336783

[42] Bennett V, Lorenzo DN. Spectrin- and ankyrin-based membrane domains and the evolution of vertebrates. Curr Top Membr. 2013;72: 1–37. doi: 10.1016/B978-0-12-417027-8.00001-5 24210426 10.1016/B978-0-12-417027-8.00001-5

[43] Lee A, Morrow JS, Fowler VM. Caspase remodeling of the spectrin membrane skeleton during lens development and aging. J Biol Chem. 2001;276: 20735–20742. doi: 10.1074/jbc.M009723200 11278555 10.1074/jbc.M009723200

[44] Cheng C, Nowak RB, Fowler VM. The lens actin filament cytoskeleton: Diverse structures for complex functions. Exp Eye Res. 2017;156: 58–71. doi: 10.1016/j.exer.2016.03.005 26971460 10.1016/j.exer.2016.03.005 PMC5018247

[45] Brinegar AE, Cooper TA. Roles for RNA-binding proteins in development and disease. Brain Res. 2016;1647: 1–8. doi: 10.1016/j.brainres.2016.02.050 26972534 10.1016/j.brainres.2016.02.050 PMC5003702

[46] Dash S, Dang CA, Beebe DC, Lachke SA. Deficiency of the RNA binding protein caprin2 causes lens defects and features of peters anomaly. Dev Dyn. 2015;244: 1313–1327. doi: 10.1002/dvdy.24303 26177727 10.1002/dvdy.24303 PMC4586403

[47] Millard SS, Vidal A, Markus M, Koff A. A U-rich element in the 5’ untranslated region is necessary for the translation of p27 mRNA. Mol Cell Biol. 2000;20: 5947–5959. 10913178 10.1128/mcb.20.16.5947-5959.2000 PMC86072

[48] Göpfert U, Kullmann M, Hengst L. Cell cycle-dependent translation of p27 involves a responsive element in its 5’-UTR that overlaps with a uORF. Hum Mol Genet. 2003;12: 1767–1779. 12837699 10.1093/hmg/ddg177

[49] Jiang H, Coleman J, Miskimins R, Srinivasan R, Miskimins WK. Cap-independent translation through the p27 5’-UTR. Nucleic Acids Res. 2007;35: 4767–4778. doi: 10.1093/nar/gkm512 17617641 10.1093/nar/gkm512 PMC1950543

[50] Kullmann M, Göpfert U, Siewe B, Hengst L. ELAV/Hu proteins inhibit p27 translation via an IRES element in the p27 5’UTR. Genes Dev. 2002;16: 3087–3099. doi: 10.1101/gad.248902 12464637 10.1101/gad.248902 PMC187493

[51] Beaumont C, Leneuve P, Devaux I, Scoazec JY, Berthier M, Loiseau MN, et al Mutation in the iron responsive element of the L ferritin mRNA in a family with dominant hyperferritinaemia and cataract. Nat Genet. 1995;11: 444–446. doi: 10.1038/ng1295-444 7493028 10.1038/ng1295-444

[52] Iakova P, Wang G-L, Timchenko L, Michalak M, Pereira-Smith OM, Smith JR, et al Competition of CUGBP1 and calreticulin for the regulation of p21 translation determines cell fate. EMBO J. 2004;23: 406–417. doi: 10.1038/sj.emboj.7600052 14726956 10.1038/sj.emboj.7600052 PMC1271759

[53] Gareau C, Fournier M-J, Filion C, Coudert L, Martel D, Labelle Y, et al p21(WAF1/CIP1) upregulation through the stress granule-associated protein CUGBP1 confers resistance to bortezomib-mediated apoptosis. PLoS ONE. 2011;6: e20254 doi: 10.1371/journal.pone.0020254 21637851 10.1371/journal.pone.0020254 PMC3102688

[54] Manning KS, Cooper TA. The roles of RNA processing in translating genotype to phenotype. Nat Rev Mol Cell Biol. 2017;18: 102–114. doi: 10.1038/nrm.2016.139 27847391 10.1038/nrm.2016.139 PMC5544131

[55] Kwan KM, Fujimoto E, Grabher C, Mangum BD, Hardy ME, Campbell DS, et al The Tol2kit: a multisite gateway-based construction kit for Tol2 transposon transgenesis constructs. Dev Dyn. 2007;236: 3088–3099. doi: 10.1002/dvdy.21343 17937395 10.1002/dvdy.21343

[56] Uribe RA, Gross JM. Immunohistochemistry on cryosections from embryonic and adult zebrafish eyes. CSH Protoc. 2007;2007: pdb.prot4779. 10.1101/pdb.prot4779 21357120

[57] Seritrakul P, Gross JM. Expression of the de novo DNA methyltransferases (dnmt3—dnmt8) during zebrafish lens development. Dev Dyn. 2014;243: 350–356. doi: 10.1002/dvdy.24077 24123478 10.1002/dvdy.24077

[58] Reed NA, Oh DJ, Czymmek KJ, Duncan MK. An immunohistochemical method for the detection of proteins in the vertebrate lens. J Immunol Methods. 2001;253: 243–252. 11384685 10.1016/s0022-1759(01)00374-x

[59] Anand D, Agrawal S, Siddam A, Motohashi H, Yamamoto M, Lachke SA. An integrative approach to analyze microarray datasets for prioritization of genes relevant to lens biology and disease. Genom Data. 2015;5: 223–227. doi: 10.1016/j.gdata.2015.06.017 26185746 10.1016/j.gdata.2015.06.017 PMC4500531

[60] Le Tonquèze O, Gschloessl B, Namanda-Vanderbeken A, Legagneux V, Paillard L, Audic Y. Chromosome wide analysis of CUGBP1 binding sites identifies the tetraspanin CD9 mRNA as a target for CUGBP1-mediated down-regulation. Biochem Biophys Res Commun. 2010;394: 884–889. doi: 10.1016/j.bbrc.2010.03.020 20227387 10.1016/j.bbrc.2010.03.020

