# Supplementary figures and images for: The RNA-binding protein Celf1 post-transcriptionally regulates p27Kip1 and Dnase2b to control fiber cell nuclear degradation in lens development
Source: PLoS Genet. 2018 Mar 22;14(3):e1007278. doi: 10.1371/journal.pgen.1007278 (PMC5889275; doi:10.1371/journal.pgen.1007278)

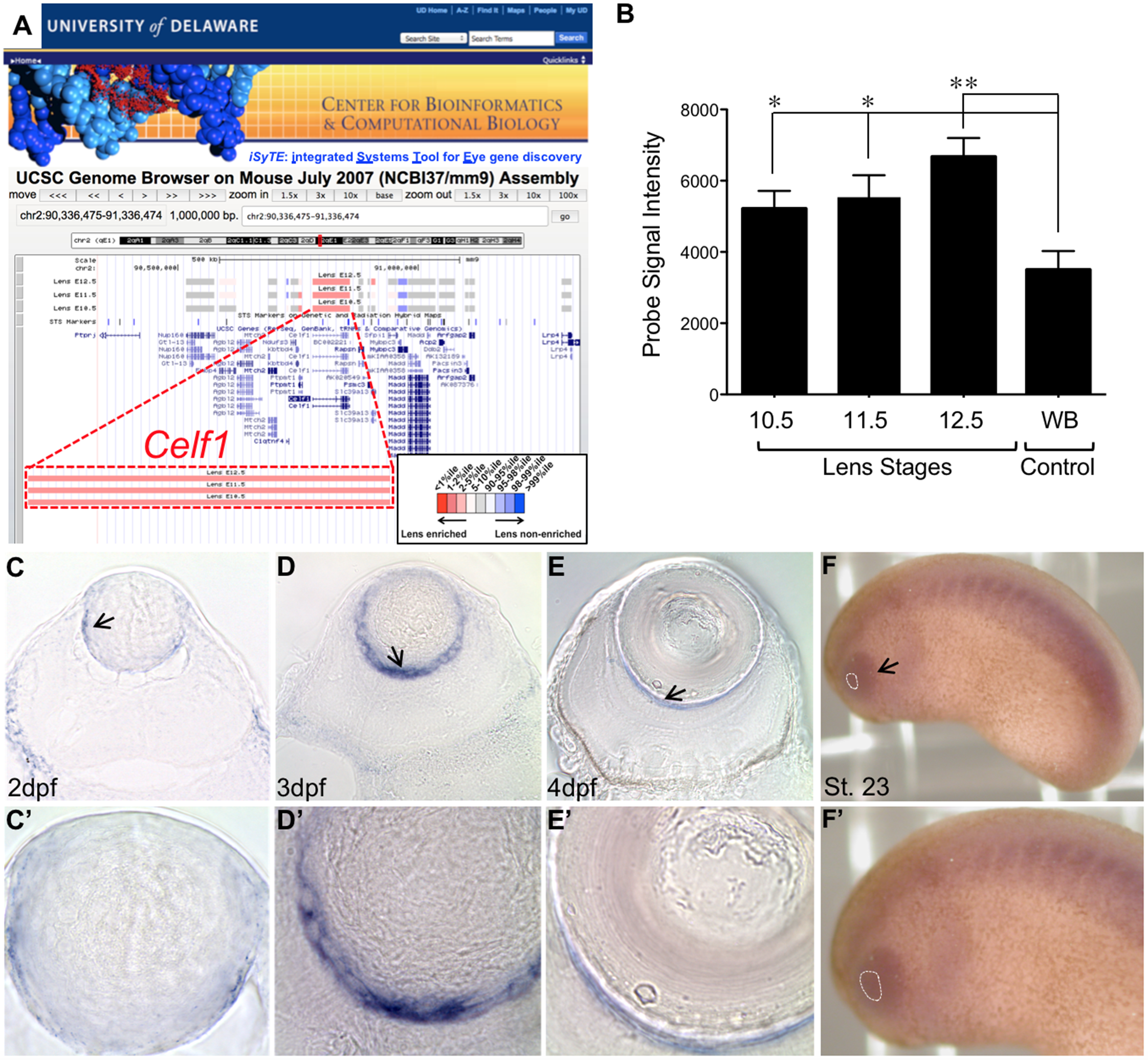

Supplement: S1 Fig — (A) iSyTE identifies Celf1 as a highly lens-enriched gene in lens development. Lens enrichment extent is indicated by differing red color intensities in the heat-map. (B) Analysis of iSyTE lens microarray datasets from mouse embryonic stages 10.5, 11.5 and 12.5 shows highly enriched expression of Celf1 compared to whole embryonic body tissue (WB) control. Celf1 microarray probe binding fluorescent signal intensity is represented on the Y-axis and the different mouse embryonic lens stages are shown on the X-axis. (C) At 2dpf (day post fertilization), (D) 3dpf and (E) 4dpf, zebrafish lenses exhibit expression of celf1 mRNA in the transition zone (arrow in C) and in later stages in the posterior region (arrows in D and E). (C’ to E’) High-magnification of C to E. (F) In Xenopus laevis, celf1 mRNA expression is observed from early developmental stage (St. 23) in the eye region (arrow; lens area indicated by broken white line). (F’) High-magnification of F. Lens area is indicated by broken white line. Asterisks in B represents a p-value less than 0.05. (TIFF) [file pgen.1007278.s001.tiff]

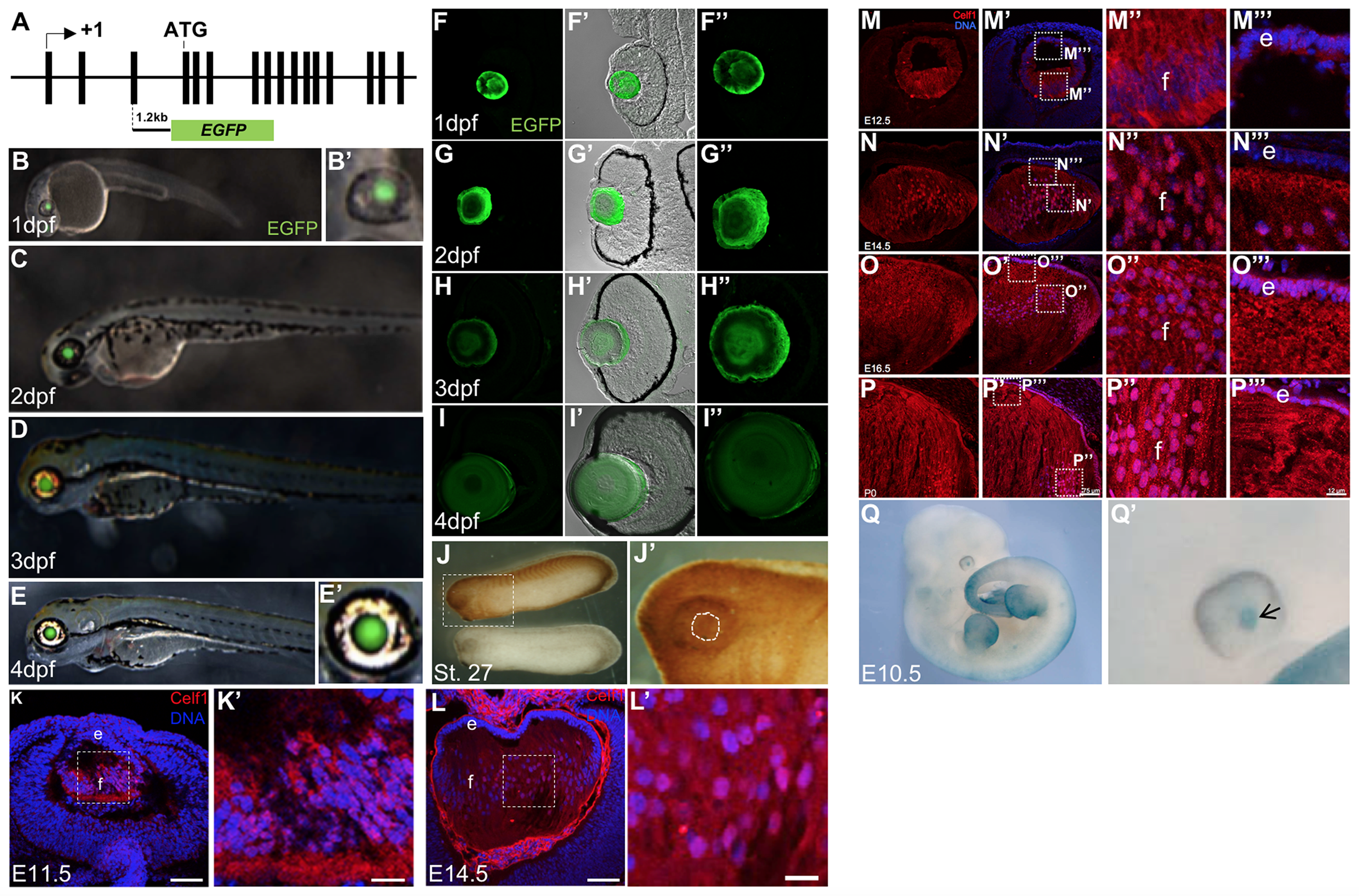

Supplement: S2 Fig — (A) Schematic of the zebrafish celf1 gene (not drawn to scale) shows the location of the ~1.2kb potential enhancer in the genomic region upstream of the start codon (which is located in exon 4). This ~1.2kb celf1 genomic region is fused to EGFP in the plasmid construct that is used in the reporter assays. (B to E’) Lens-specific expression of EGFP in zebrafish indicates strong celf1 enhancer activity at (B, B’) 1dpf, (C) 2dpf, (D) 3dpf and (E, E’) 4dpf. (F to I”) Transverse sections of zebrafish eye exhibit high EGFP expression at (F to F”) 1dpf, (G to G”) 2dpf, (H to H”) 3dpf and (I to I”) 4dpf. (J) In Xenopus laevis, at St. 27, high celf1 protein expression is present. (J’) High-magnification of dotted line area in J. Dotted-line circle shows lens region. (K and L) In mouse lens, high Celf1 protein expression is detected by a mouse monoclonal Celf1 antibody at stages E11.5 and E14.5. Fiber cells (f) and epithelium (e). (K’ and L’) High-magnification of dotted-line area in K and L, respectively. (M-P”‘) In mouse lens, rabbit Celf1 antibody detects Celf1 protein in lens development at stages E12.5, E14.5, E16.5 and P0. High magnification in dotted area is shown. In early stages, Celf1 protein is detected predominantly in fiber cells (f), and at later stages, while it retains high fiber cell expression, it is also detected in the epithelium (e). (Q) Celf1lacZKI/+ mouse reporter analysis reveals β-galactosidase activity in the lens at embryonic stage E11.5, indicative of endogenous Celf1 promoter/enhancer driven gene expression. (Q’) High-magnification of eye region in M’ shows high β-galactosidase activity in the lens (arrow). Scale bar in K and L is 75 μm while in K’ and L’ is 12 μm. (TIFF) [file pgen.1007278.s002.tiff]

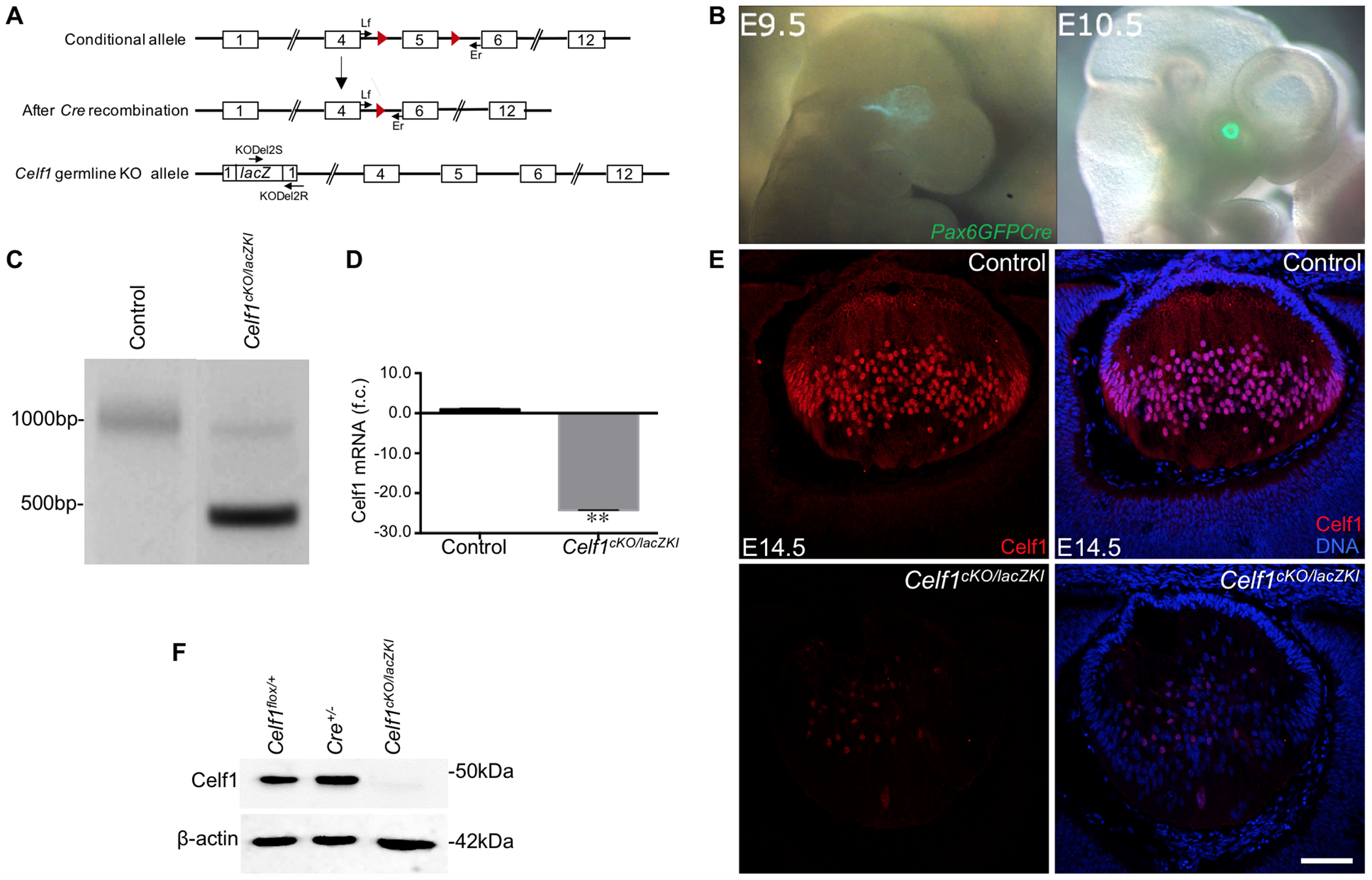

Supplement: S3 Fig — (A) Schematic representation of targeting strategy to generate Celf1 conditional knockout (Celf1cKO/cKO) and Celf1 compound conditional (Celf1cKO/lacZKI) mice. The “conditional allele” represents Celf1 floxed allele wherein, exon five is flanked by loxP (red arrowheads). The “after Cre recombination allele” shows the rearranged Celf1 allele after Cre mediated exon five deletion. The “Celf1 germline KO allele” (Celf1lacZKI) represents the Celf1 germline targeted allele that has the lacZ cassette inserted in exon one as previously described [11]. Black arrows indicate position of genotyping primers. (B) Pax6GFPCre transgenic mouse line carries a GFP-Cre fusion gene driven by the Pax6 P0 promoter 3.9-kb upstream region and show GFP-Cre expression early in lens development starting in the lens placode stage at embryonic stage E9.5. Strong GFP-Cre is observed in the lens vesicle at E10.5. (C) PCR analysis confirms the deletion of the floxed exon five in lens DNA obtained from Celf1cKO/lacZKI mice. The Celf1 lacZ knock-in allele is as previously described [11]. (D) RT-qPCR analysis confirms significantly reduced (~25-fold) Celf1 mRNA levels in P0 Celf1cKO/lacZKI lens. (E) Compared to control, immunofluorescence analysis with and without Draq5 staining of DNA shows the near absence of Celf1 protein in Celf1cKO/lacZKI lens at E14.5. (F) Western blot analysis shows the absence of Celf1 protein in Celf1cKO/lacZKI lenses at P30, confirming Celf1 deletion in the mouse lens. Asterisks in D represent a p-value of less than 0.005. Scale bar in E is 12 μm. (TIFF) [file pgen.1007278.s003.tiff]

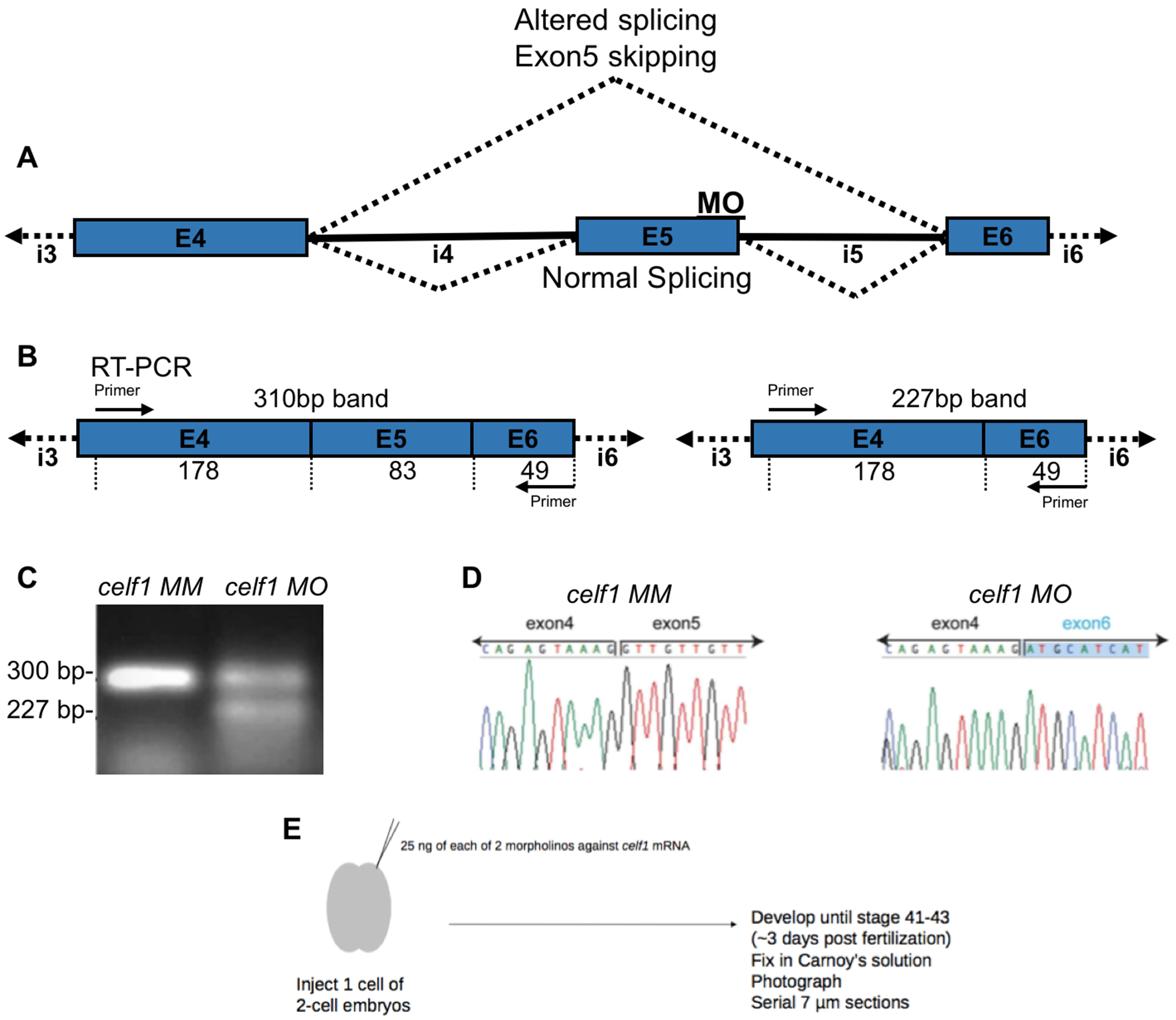

Supplement: S4 Fig — (A) Schematic representation of normal and altered splicing of celf1 in zebrafish. celf1 morphants (celf1 MO) were generated by using splice altering morpholinos to knockdown celf1 in zebrafish. (B) Schematic representation of PCR strategy to detect normal (310 bp) and altered splicing (227 bp) in zebrafish. (C) RT-PCR showing a 310 bp band indicating normal splicing in celf1 controls (celf1 MM) and a 227 bp band in the celf1-knockdown (celf1 MO) embryos confirming splice altering activity in zebrafish morphants. (D) Sequencing data confirms the exclusion of exon five in zebrafish celf1 MO embryos but not in controls. (E) In X. laevis, celf1 KD animals were generated by injecting morpholinos against celf1 (see methods) in one of the cells of the embryos at the two-cell-stage, as described previously [21]. (TIFF) [file pgen.1007278.s004.tiff]

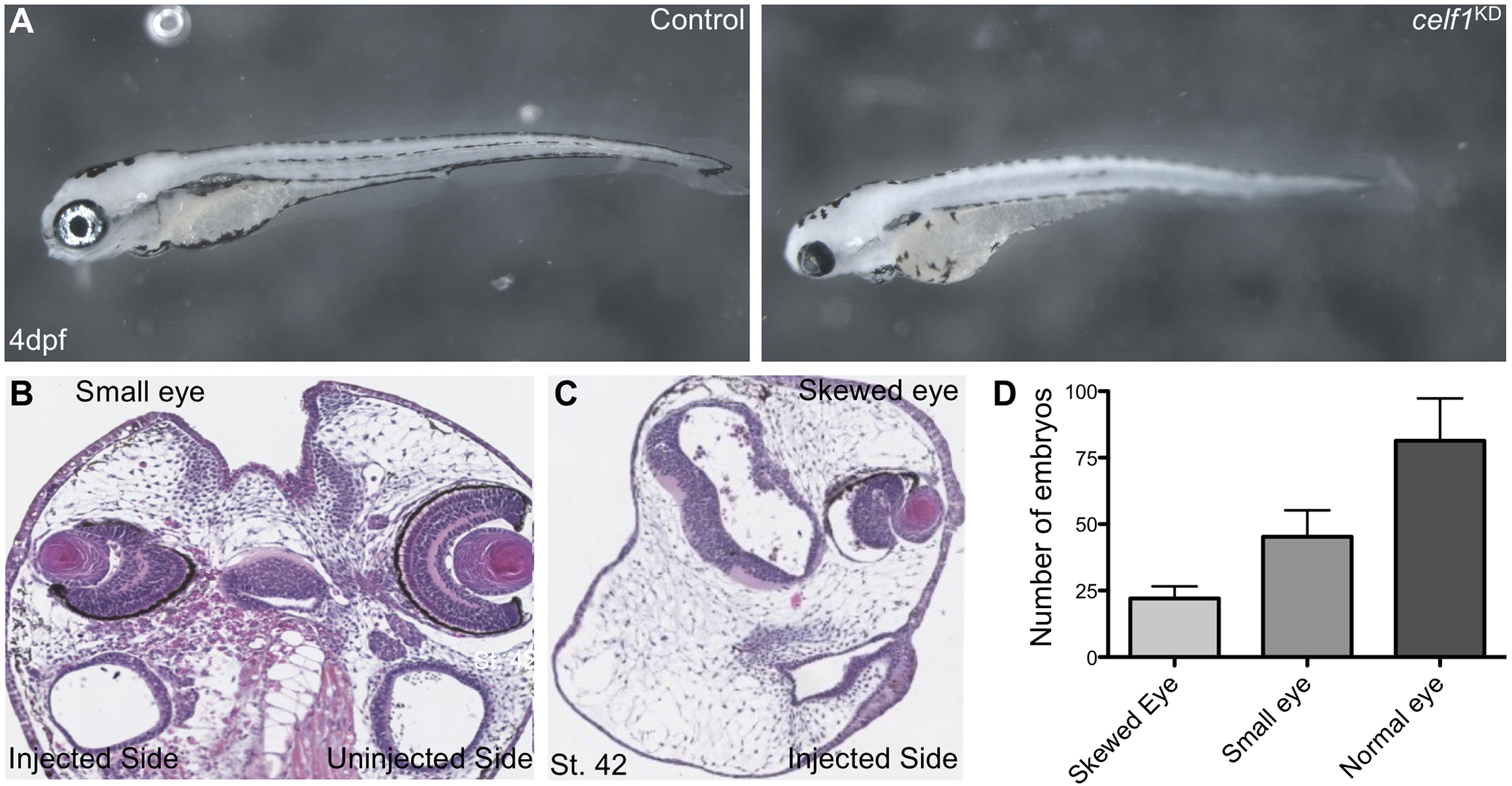

Supplement: S5 Fig — (A) celf1 knockdown (KD) in zebrafish results in microphthalmia and clouding of the eye by 4dpf. (B and C) Histological analysis of X. laevis morphants at St. 42. celf1-knockdown (morpholino injected side) shows various eye defects such as (B) small eye and (C) skewed eye, that are absent in the control uninjected side. (D) Graph representing the distribution of the observed eye defects in X. laevis celf1 morphants from three independent experiments. (TIFF) [file pgen.1007278.s005.tiff]

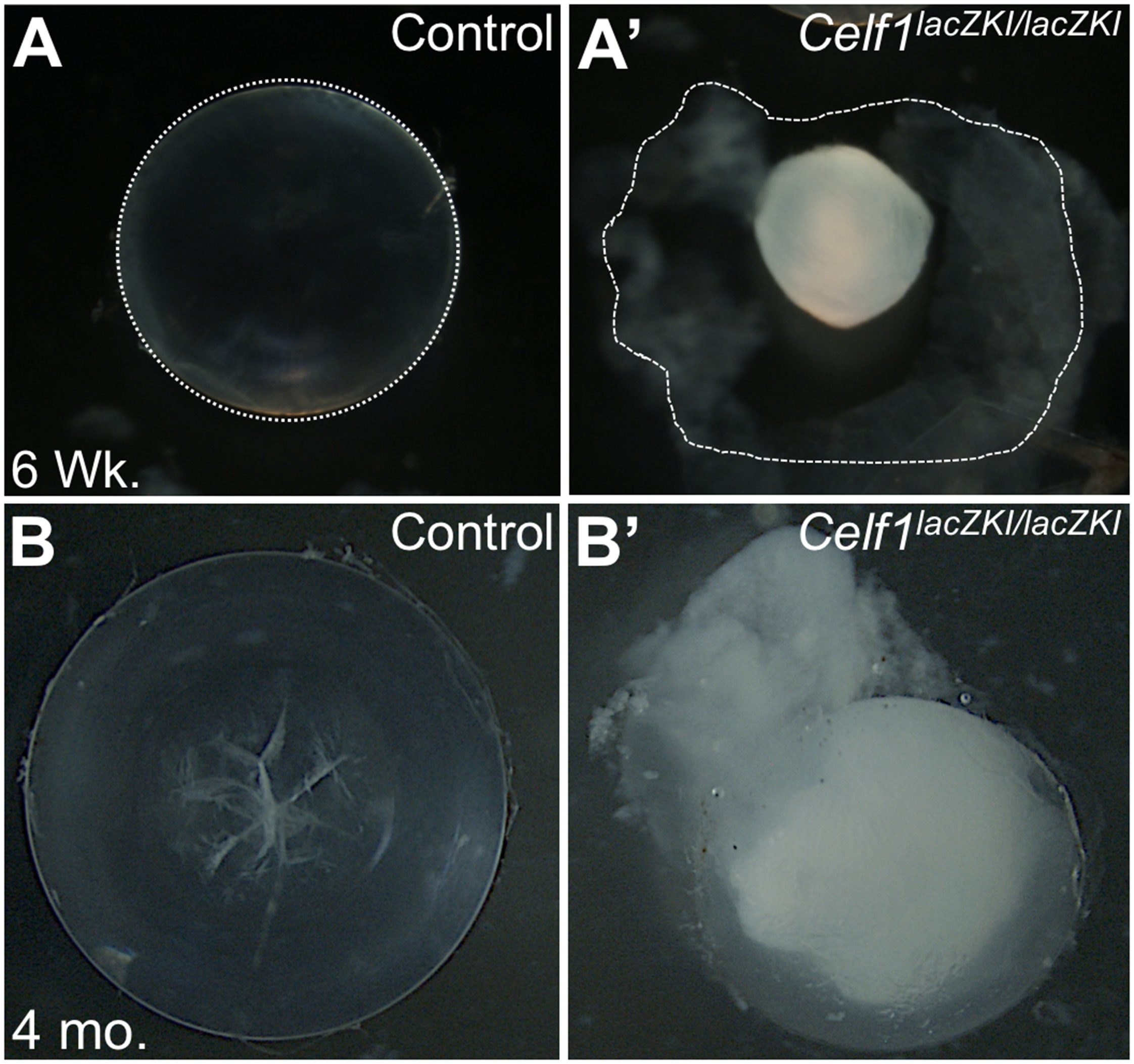

Supplement: S6 Fig — (A to B’) Six weeks (A, A’) and four month (B, B’) aged Celf1lacZKI/lacZKI lenses shows severe cataracts and disruption of the lens tissue compared to control. In A’, the dotted area represents the disintegrated lens tissue around the remaining lens core in Celf1lacZKI/lacZKI mice. (C to C’) Histological analysis of Celf1cKO/cKO lens exhibits slightly delayed fiber cell elongation compared to the control lens at stage E12.5. Scale bars in C, C’ are 100 μm. (TIFF) [file pgen.1007278.s006.tiff]

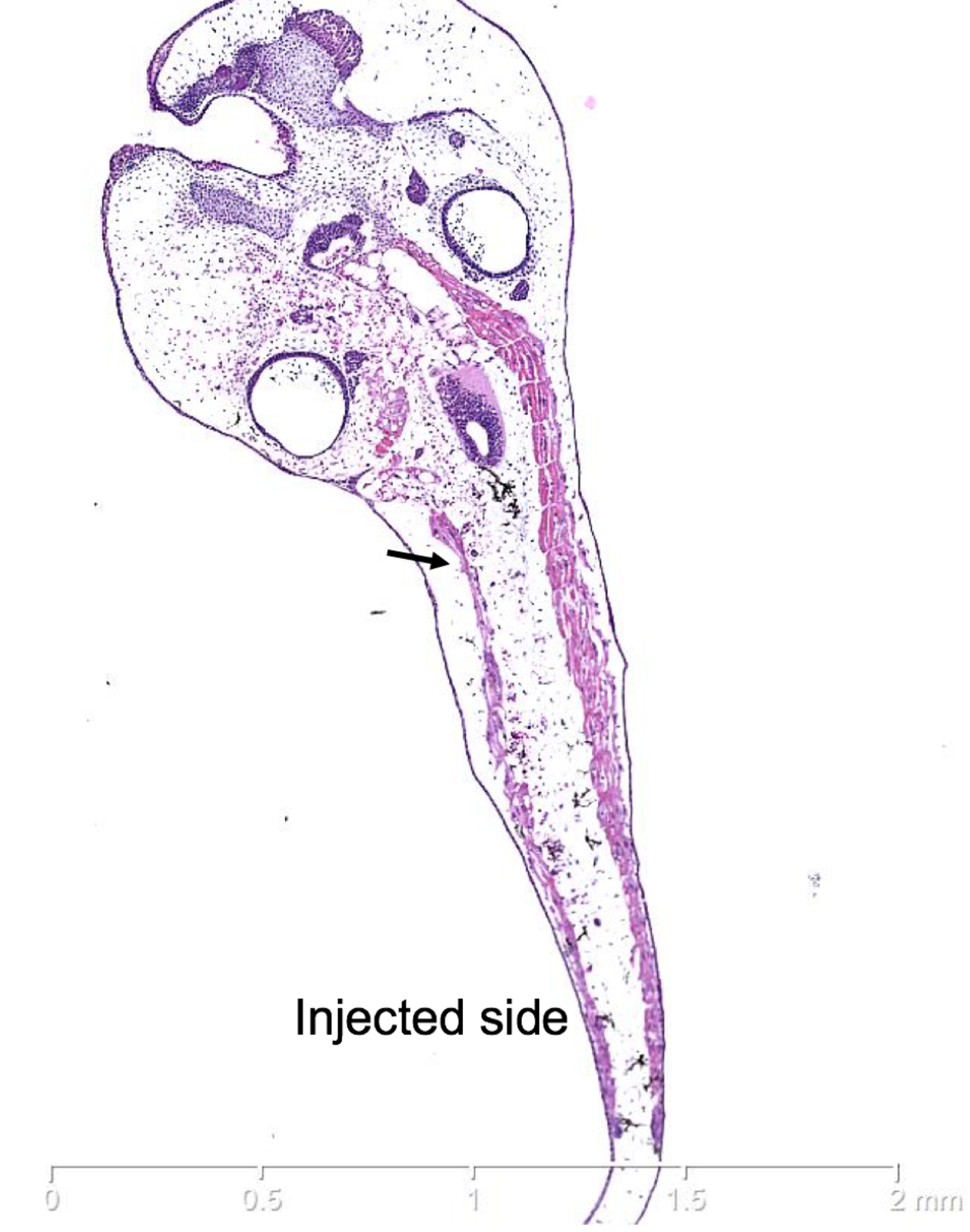

Supplement: S7 Fig — Previously, Xenopus celf1 morphants have been described to have defective somite segmentation. In our present study on Xenopus, we also observe these defects. The morpholino injected side (left) of Xenopus embryos (St. 42) shows somite segmentation defects (arrow). (TIFF) [file pgen.1007278.s007.tiff]

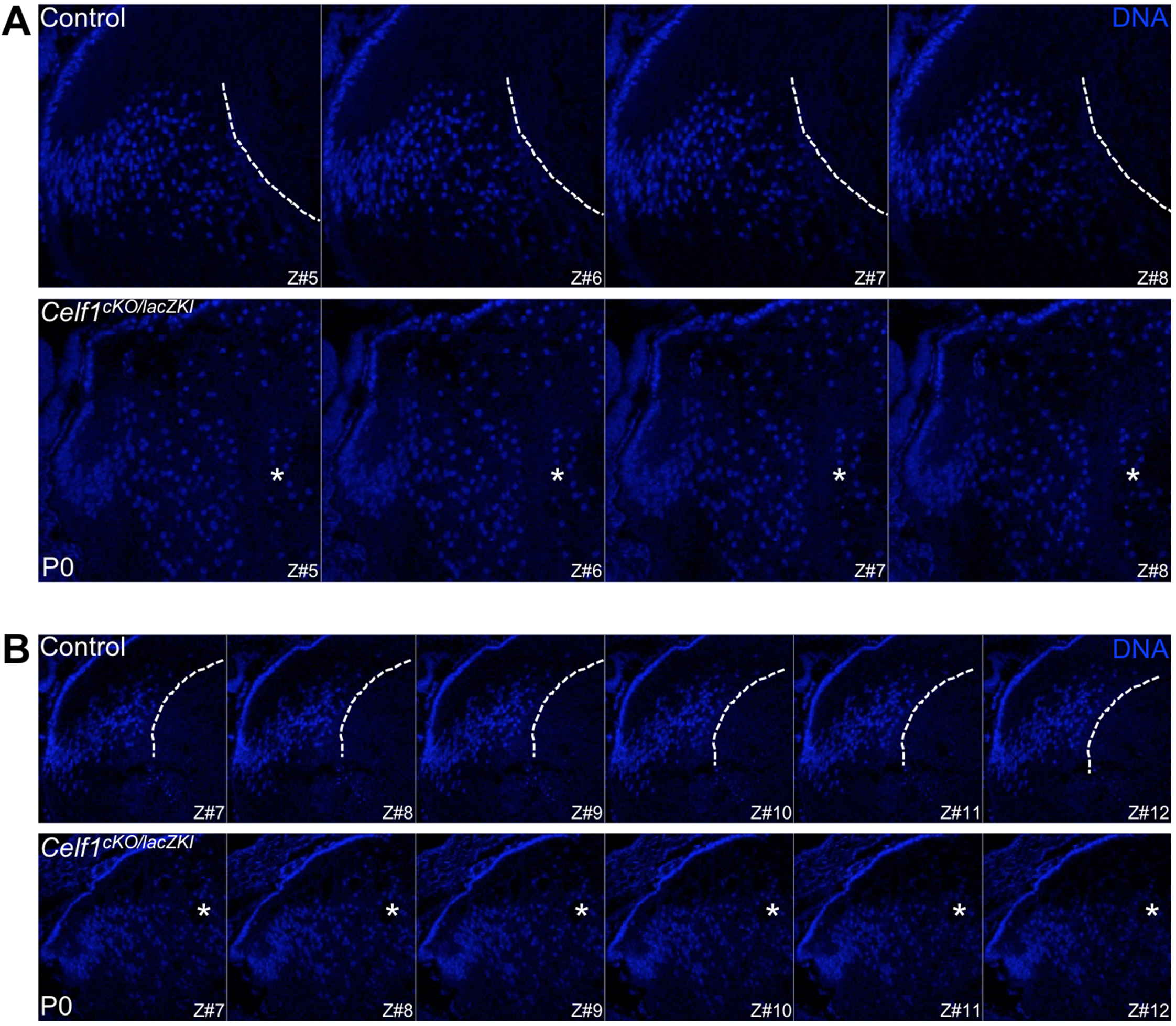

Supplement: S8 Fig — (A) Z-stack serial imaging analysis (serial slices#5 through 8 in a series of 10) demonstrates that Celf1cKO/cKO mouse lens at stage P0 exhibits presence of nuclei (asterisk) in centrally located fiber cells compared to control mouse lens which shows a distinct nuclei-free zone in this fiber cell region (broken white line). (B) Z-stack serial imaging analysis (serial slices#7 through 12 in a series of 22) demonstrates that Celf1cKO/cKO mouse lens at stage P0 exhibits presence of nuclei (asterisk) in centrally located fiber cells compared to control mouse lens which shows a distinct nuclei-free zone in this fiber cell region (broken white line). (TIFF) [file pgen.1007278.s008.tiff]

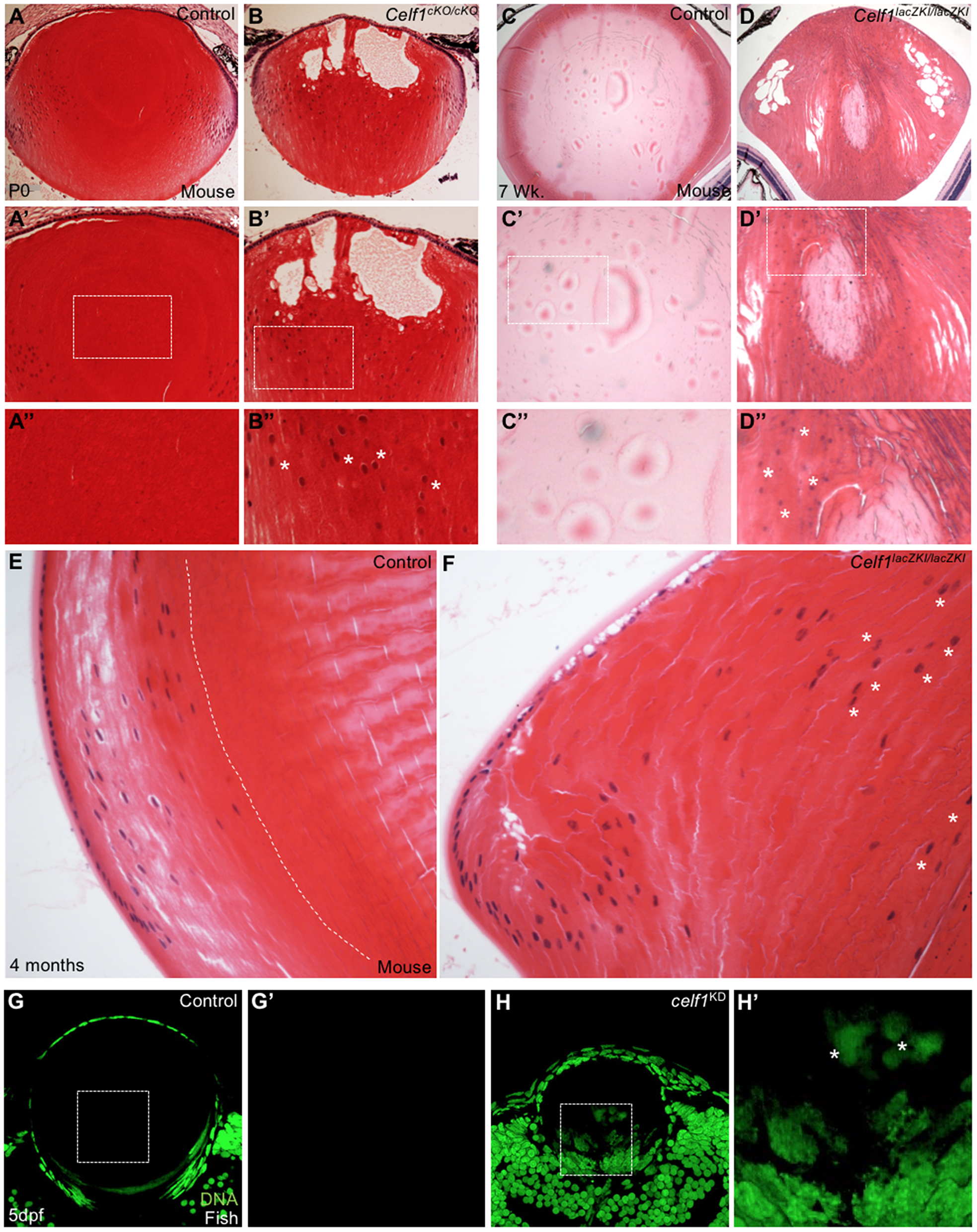

Supplement: S9 Fig — (A to B”) In mice, compared to control, Celf1cKO/cKO lens at stage P0 exhibits fiber cell nuclear degradation defects. Note the abnormal presence of nuclei (asterisks) in the central region of the fiber cells. A’ to B” are higher magnification images of A and B, respectively. (C to D”) Compared to control, Celf1lacZKI/lacZKI lens at seven-week age continue to exhibit nuclear degradation defects. Note the abnormal presence of nuclei (asterisks) in the central fiber cell region. C’ to D” are higher magnification images of C and D, respectively. (E and F) Compared to control where a clear nuclear free zone is visible (broken white line), Celf1lacZKI/lacZKI mouse lens at 4 months continue to exhibit nuclear degradation defects. Note the abnormal presence of nuclei (asterisks) in the central fiber cell region. (G to H’) Compared to control, zebrafish celf1 KD lens exhibits nuclear degradation defects at stage 5dpf. Note the abnormal presence of nuclei (asterisks) in the central fiber cell region. E’ and F’ are high-magnification of the dotted-line area in E and F. (TIFF) [file pgen.1007278.s009.tiff]

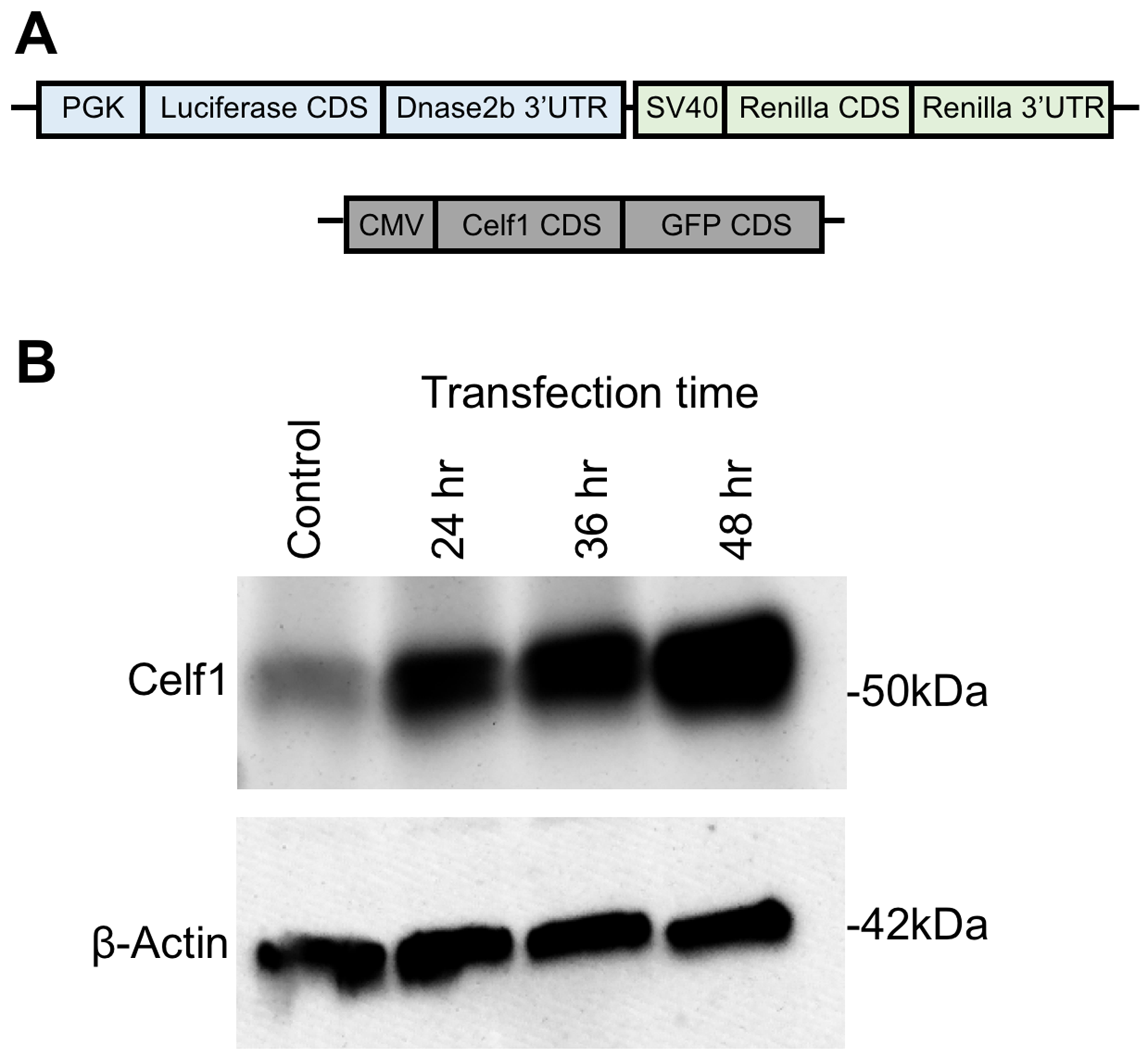

Supplement: S10 Fig — (A) Dual reporter construct of Dnase2b 3’UTR-firefly luciferase and Renilla luciferase. Celf1 CDS-GFP fusion expression construct. (B) Western blot analysis confirms over-expression of Celf1 protein at 24 hr, 36 hr, and 48 hr after the transfections compared to the control (48 hr) that was transfected with an empty vector. (TIFF) [file pgen.1007278.s010.tiff]

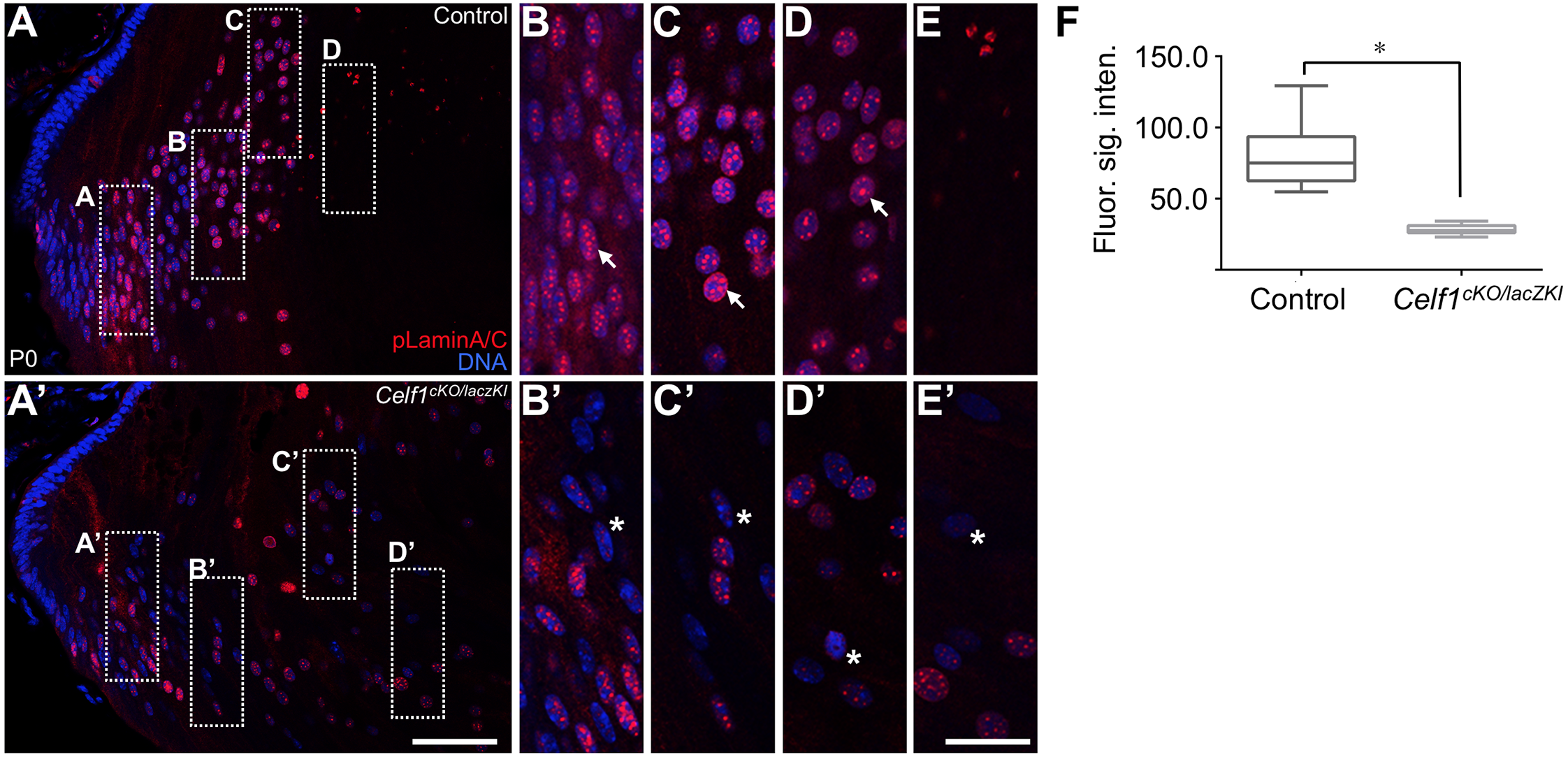

Supplement: S11 Fig — (A, A’) In Celf1cKO/lacZKI lens at stage P0, phosphorylation of Lamin A/C (pLamin A/C) is reduced compared to control. High-magnification of fiber cell regions (dotted-line areas) of control (B-E) and Celf1cKO/lacZKI (B’-E’) lens are shown. Arrows in B-D indicate high phosphorylated lamin A/C expression while its reduced expression in B’-E’ is indicated by asterisks. (F) In mouse stage P0 lens, quantification of the immunofluorescence signals shows significantly reduced Lamin A/C protein levels in Celf1cKO/LacZKI lens compared to control. Asterisk in F represents a p-value <0.05. Scale bar represents 75 μm. (TIFF) [file pgen.1007278.s011.tiff]

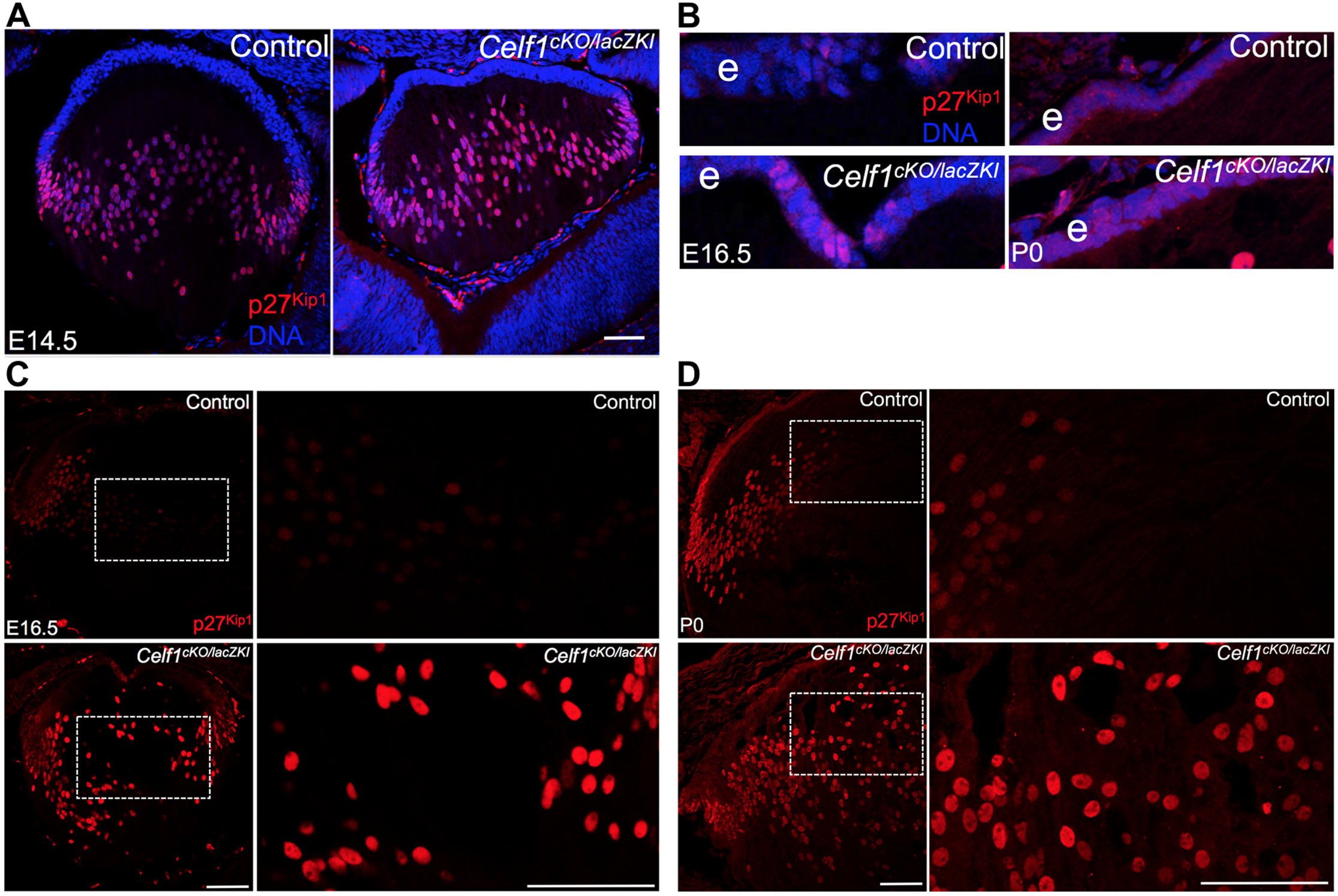

Supplement: S12 Fig — (A) At E14.5, the p27Kip1 protein levels are reduced in central fiber region in the control lens–as expected, while in the Celf1cKO/lacZKI lens, p27Kip1 protein levels in the central fiber region are elevated. (B) At E16.5, Celf1cKO/lacZKI lens epithelium exhibits elevated p27Kip1 protein in a few nuclei compared to control, but this difference was not observed at stage P0. (C, D) At stages E16.5 and P0, both control and Celf1cKO/lacZKI lenses show p27Kip1 protein expression in the transition zone and in cortical fiber cells. However, p27Kip1 protein expression is distinctly elevated in the centrally located fiber cells in Celf1cKO/lacZKI lenses compared to control. Broken white line areas of fiber cell regions is shown at high-magnification. Scale bar represents 75 μm. (TIFF) [file pgen.1007278.s012.tiff]

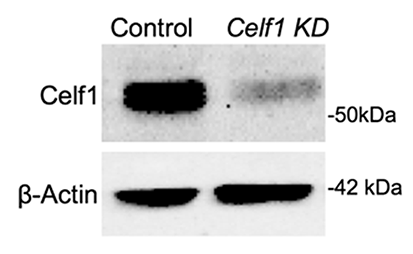

Supplement: S13 Fig — Western blot analysis confirms the reduction of Celf1 protein in the lenti-viral-based stable Celf1 knockdown lens cell line 21EM15, compared to control. (TIFF) [file pgen.1007278.s013.tiff]

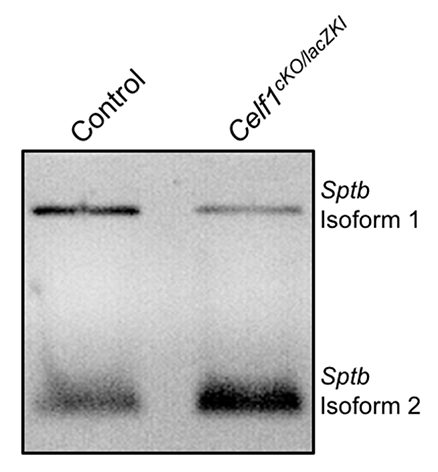

Supplement: S14 Fig — RT-PCR analysis indicates that the high-abundant Beta-spectrin (Sptb) isoform (isoform 1 (ENSMUST00000021458)) is reduced, while the low-abundant isoform (isoform 2 (ENSMUST00000166101)) is abnormally elevated in Celf1cKO/lacZKI lenses. Amplicon sizes of Sptb isoform 1 and isoform 2 are 681 and 121 base-pairs, respectively. (TIFF) [file pgen.1007278.s014.tiff]
